# Cardiovascular Risk Factors Associated With Venous Thromboembolism

**DOI:** 10.1001/jamacardio.2018.4537

**Published:** 2019-01-16

**Authors:** John Gregson, Stephen Kaptoge, Thomas Bolton, Lisa Pennells, Peter Willeit, Stephen Burgess, Steven Bell, Michael Sweeting, Eric B. Rimm, Christopher Kabrhel, Bengt Zöller, Gerd Assmann, Vilmundur Gudnason, Aaron R. Folsom, Volker Arndt, Astrid Fletcher, Paul E. Norman, Børge G. Nordestgaard, Akihiko Kitamura, Bakhtawar K. Mahmoodi, Peter H. Whincup, Matthew Knuiman, Veikko Salomaa, Christa Meisinger, Wolfgang Koenig, Maryam Kavousi, Henry Völzke, Jackie A. Cooper, Toshiharu Ninomiya, Edoardo Casiglia, Beatriz Rodriguez, Yoav Ben-Shlomo, Jean-Pierre Després, Leon Simons, Elizabeth Barrett-Connor, Cecilia Björkelund, Marlene Notdurfter, Daan Kromhout, Jackie Price, Susan E. Sutherland, Johan Sundström, Jussi Kauhanen, John Gallacher, Joline W. J. Beulens, Rachel Dankner, Cyrus Cooper, Simona Giampaoli, Jason F. Deen, Agustín Gómez de la Cámara, Lewis H. Kuller, Annika Rosengren, Peter J. Svensson, Dorothea Nagel, Carlos J. Crespo, Hermann Brenner, Juan R. Albertorio-Diaz, Robert Atkins, Eric J. Brunner, Martin Shipley, Inger Njølstad, Deborah A. Lawlor, Yvonne T. van der Schouw, Randi Marie Selmer, Maurizio Trevisan, W. M. Monique Verschuren, Philip Greenland, Sylvia Wassertheil-Smoller, Gordon D. O. Lowe, Angela M. Wood, Adam S. Butterworth, Simon G. Thompson, John Danesh, Emanuele Di Angelantonio, Tom Meade

**Affiliations:** 1 London School of Hygiene and Tropical Medicine, London, United Kingdom; 2 MRC/BHF Cardiovascular Epidemiology Unit, Department of Public Health and Primary Care, University of Cambridge, Cambridge, United Kingdom; 3 National Institute for Health Research Blood and Transplant Research Unit in Donor Health and Genomics, University of Cambridge, Cambridge, United Kingdom; 4 Medical University of Innsbruck, Innsbruck, Austria; 5 MRC Biostatistics Unit, Cambridge University, Cambridge, United Kingdom; 6 Harvard T. H. Chan School of Public Health, Boston, Massachusetts; 7 Massachusetts General Hospital, Boston; 8 Department of Clinical Sciences, Lund University, Malmö, Sweden; 9 Assmann Foundation for Prevention, Münster, Germany; 10 Icelandic Heart Association, Kópavogur, Iceland; 11 University of Minnesota School of Public Health, Minneapolis; 12 Division of Clinical Epidemiology and Aging Research, German Cancer Research Center (DKFZ), Heidelberg, Germany; 13 University of Western Australia, Perth, Western Australia, Australia; 14 Department of Clinical Biochemistry, Herlev and Gentofte Hospital, Copenhagen University Hospital, Copenhagen, Denmark; 15 Faculty of Health and Medical Sciences, University of Copenhagen, Copenhagen, Denmark; 16 Copenhagen City Heart Study, Frederiksberg Hospital, Copenhagen University Hospital, Copenhagen, Denmark; 17 Osaka University, Osaka, Japan; 18 University Medical Center Groningen, University of Groningen, Groningen, the Netherlands; 19 St George’s, University of London, London, United Kingdom; 20 National Institute for Health and Welfare, Helsinki, Finland; 21 Ludwig Maximilian University of Munich, Munich, Germany; 22 Helmholtz Zentrum München, German Research Center for Environmental Health, Neuherberg, Germany; 23 Deutsches Herzzentrum München, Technische Universität München, Munich, Germany; 24 German Centre for Cardiovascular Research (DZHK), partner site Munich Heart Alliance, Munich, Germany; 25 Department of Internal Medicine II–Cardiology, University of Ulm Medical Center, Ulm, Germany; 26 Erasmus University Medical Center, Erasmus University, Rotterdam, the Netherlands; 27 University of Greifswald, Greifswald, Germany; 28 UCL Medical School, University College London, London, United Kingdom; 29 Kyushu University, Fukuoka, Japan; 30 University of Padova, Padua, Italy; 31 University of Hawaii, Honolulu; 32 Population Health Science, Bristol Medical School, University of Bristol, Bristol, United Kingdom; 33 Institute of Nutraceuticals and Functional Foods, Université Laval, Quebec, Quebec, Canada; 34 The University of New South Wales, Sydney, New South Wales, Australia; 35 University of California, San Diego; 36 University of Gothenburg, Gothenburg, Sweden; 37 Department of Internal Medicine, Bruneck Hospital, Bruneck, Italy; 38 University of Edinburgh, Edinburgh, United Kingdom; 39 Medical University of South Carolina, Charleston; 40 Institute of Public Health and Clinical Nutrition, University of Eastern Finland, Kuopio, Finland; 41 VU University Medical Center Amsterdam, Amsterdam, the Netherlands; 42 Julius Center for Health Sciences and Primary Care, University Medical Center Utrecht, Utrecht University, Utrecht, the Netherlands; 43 Tel Aviv University, Tel Aviv, Israel; 44 MRC Lifecourse Epidemiology Unit, University of Southampton, Southampton, United Kingdom; 45 National Institute of Health (ISS), Rome, Italy; 46 Center of Health Equity, Diversity and Inclusion, University of Washington School of Medicine, Seattle; 47 Clinical Research and Clinical Trials Unit, Plataforma de Innovación en Tecnologías Médicas y Sanitarias, Madrid, Spain; 48 University of Pittsburgh Graduate School of Public Health, Pittsburgh, Pennsylvania; 49 Portland State University, Portland, Oregon; 50 US Centers for Disease Control and Prevention, Atlanta, Georgia; 51 Monash University, Melbourne, Victoria, Australia; 52 Department of Epidemiology and Public Health, University College London, London, United Kingdom; 53 Norwegian Institute of Public Health, Oslo, Norway; 54 MRC Integrative Epidemiology Unit at the University of Bristol, Bristol, United Kingdom; 55 CUNY School of Medicine, City University of New York, New York; 56 Centre for Nutrition, Prevention and Health Services, National Institute for Public Health and the Environment (RIVM), Bilthoven, the Netherlands; 57 Feinberg School of Medicine, Northwestern University, Chicago, Illinois; 58 Albert Einstein College of Medicine, New York, New York; 59 Institute of Cardiovascular and Medical Sciences, University of Glasgow, Glasgow, United Kingdom

## Abstract

**Importance:**

It is uncertain to what extent established cardiovascular risk factors are associated with venous thromboembolism (VTE).

**Objective:**

To estimate the associations of major cardiovascular risk factors with VTE, ie, deep vein thrombosis and pulmonary embolism.

**Design, Setting, and Participants:**

This study included individual participant data mostly from essentially population-based cohort studies from the Emerging Risk Factors Collaboration (ERFC; 731 728 participants; 75 cohorts; years of baseline surveys, February 1960 to June 2008; latest date of follow-up, December 2015) and the UK Biobank (421 537 participants; years of baseline surveys, March 2006 to September 2010; latest date of follow-up, February 2016). Participants without cardiovascular disease at baseline were included. Data were analyzed from June 2017 to September 2018.

**Exposures:**

A panel of several established cardiovascular risk factors.

**Main Outcomes and Measures:**

Hazard ratios (HRs) per 1-SD higher usual risk factor levels (or presence/absence). Incident fatal outcomes in ERFC (VTE, 1041; coronary heart disease [CHD], 25 131) and incident fatal/nonfatal outcomes in UK Biobank (VTE, 2321; CHD, 3385). Hazard ratios were adjusted for age, sex, smoking status, diabetes, and body mass index (BMI).

**Results:**

Of the 731 728 participants from the ERFC, 403 396 (55.1%) were female, and the mean (SD) age at the time of the survey was 51.9 (9.0) years; of the 421 537 participants from the UK Biobank, 233 699 (55.4%) were female, and the mean (SD) age at the time of the survey was 56.4 (8.1) years. Risk factors for VTE included older age (ERFC: HR per decade, 2.67; 95% CI, 2.45-2.91; UK Biobank: HR, 1.81; 95% CI, 1.71-1.92), current smoking (ERFC: HR, 1.38; 95% CI, 1.20-1.58; UK Biobank: HR, 1.23; 95% CI, 1.08-1.40), and BMI (ERFC: HR per 1-SD higher BMI, 1.43; 95% CI, 1.35-1.50; UK Biobank: HR, 1.37; 95% CI, 1.32-1.41). For these factors, there were similar HRs for pulmonary embolism and deep vein thrombosis in UK Biobank (except adiposity was more strongly associated with pulmonary embolism) and similar HRs for unprovoked vs provoked VTE. Apart from adiposity, these risk factors were less strongly associated with VTE than CHD. There were inconsistent associations of VTEs with diabetes and blood pressure across ERFC and UK Biobank, and there was limited ability to study lipid and inflammation markers.

**Conclusions and Relevance:**

Older age, smoking, and adiposity were consistently associated with higher VTE risk.

## Key Points

**Question:**

To what extent are established cardiovascular risk factors associated with risk of venous thromboembolism (VTE)?

**Findings:**

In this analysis of individual participant data from the Emerging Risk Factors Collaboration and the UK Biobank including 1.1 million participants, among a panel of several established cardiovascular risk factors, older age, smoking, and greater adiposity were consistently associated with higher VTE risk.

**Meaning:**

There is overlap in at least some of the major population determinants of important venous and arterial thrombotic diseases.

## Teaser

This analysis of data from the Emerging Risk Factors Collaboration and the UK Biobank estimates the associations of major cardiovascular risk factors with venous thromboembolism.

## Introduction

Venous thromboembolism (VTE), consisting of deep vein thrombosis (DVT) or pulmonary embolism (PE), is a major clinical burden. Globally, there are about 10 million cases every year, and it is the third leading vascular disease after myocardial infarction and stroke.^1^ Pulmonary embolism is a manifestation of VTE and is responsible for most VTE deaths.^2^ In recent years, efforts to prevent VTE have broadened from focusing mainly on hospital-based risk factors (eg, recent prior surgery, cancer, and congestive heart failure) toward adoption of heart-healthy lifestyles.^3^ This perspective has challenged traditional views of venous and arterial thrombosis as distinct pathologies, encouraging prevention strategies that concomitantly address VTE and arterial thrombosis.^2,4^ However, there is uncertainty about the extent to which venous and arterial thrombosis share cardiovascular risk factors, as studies have reported conflicting findings.^5,6,7,8,9,10,11,12,13,14,15^ Interpretation has been complicated by the use of retrospective case-control designs, limited statistical power, and/or inability to compare VTE and arterial disease outcomes within the same cohorts.^16,17,18,19,20,21,22,23,24,25,26^

Analyzing data from more than 1.1 million participants in 76 prospective studies, we investigated associations of several established cardiovascular risk factors with the incidence of VTE outcomes. We aimed to address 2 principal questions: What are the associations of major cardiovascular risk factors with VTE outcomes (including subtypes)? How do these associations compare with those for coronary heart disease (CHD), a manifestation of arterial thrombotic disease?

## Methods

### Data Sources and Participant Inclusion

We analyzed data from the Emerging Risk Factors Collaboration (ERFC), a consortium of prospective cohort studies with information on a variety of risk factors, and the UK Biobank, a single large prospective study. Both the ERFC and UK Biobank have been described previously.^27,28^ Both data sources involve a prospective cohort study design and accessible individual participant data, enabling standardized and detailed analyses using a common protocol, including definitions for VTE and CHD outcomes. However, we conducted parallel (rather than pooled) analyses of the 2 sources because of potentially important differences in their approaches to VTE ascertainment, ie, the ERFC recorded only fatal VTE outcomes while UK Biobank recorded both fatal and nonfatal VTE outcomes, most of which were nonfatal. Information about each of the 76 studies contributing to this analysis is provided in the eAppendix in the Supplement. The study was designed and conducted by the Emerging Risk Factors Collaboration academic coordinating center, and it was approved by the Cambridgeshire Ethics Review Committee. Informed consent was obtained from participants in each of the cohorts contributing to the analysis.

Participants in the contributing studies were eligible for inclusion in the current analysis if they met all of the following criteria: (1) had recorded information on several established cardiovascular risk factors (as a minimum, information on age, sex, smoking status, history of diabetes, and body mass index [BMI]), (2) did not have a known baseline history of cardiovascular disease (CVD; defined as CHD, other heart disease, stroke, transient ischemic attack, peripheral vascular disease, or cardiovascular surgery) or VTE (defined as DVT or PE), and (3) had at least 1 year of follow-up data after baseline.

In the ERFC, only fatal VTE events were recorded. Ascertainment was based on death certificates supplemented in 56 studies by medical records, findings on autopsy, and other sources. In UK Biobank, fatal and nonfatal VTEs were ascertained through linkage with routinely collected medical records. We attempted to subcategorize VTEs as provoked and unprovoked using a pragmatic approach that required inference from routine records (eAppendix in the Supplement). Briefly, following the example of previous work,^13^ we defined VTE as provoked if, in the 90-day period preceding the VTE, the participant was recorded as having a malignant neoplasm (per cancer registry data); starting or ending a hospital episode with a main diagnosis code relating to malignant neoplasm, heart failure, infectious disease, or trauma; or having a hospital episode that included certain types of surgical procedures. The specific *International Statistical Classification of Diseases and Related Health Problems *(*ICD*) codes and *Classification of Interventions and Procedures* codes that are included in our definition are summarized in the eAppendix in the Supplement. All studies used definitions of CHD based on World Health Organization (or similar) criteria. In registering fatal outcomes, the contributing studies classified deaths according to the primary cause (or, in its absence, the underlying cause) on the basis of *ICD*-*8*, *ICD*-*9*, and *ICD*-*10* codes to at least 3 digits or according to study-specific classification systems. In the ERFC, baseline surveys were given between February 1960 and June 2008, and the date of latest follow-up was December 2015 (median, 2014 across studies); in the UK Biobank, baseline surveys were given between March 2006 and September 2010, and the date of latest follow-up was February 2016.

### Statistical Analysis

For continuous risk factors, we calculated hazard ratios (HRs) per 1-SD higher usual risk factor level. For binary risk factors, we compared presence vs absence of the factor. Cox proportional hazards regression models were adjusted for age, smoking status, history of diabetes, and BMI and stratified by study, sex, and (when appropriate) trial arm. To avoid overadjustment, we did not routinely adjust for systolic blood pressure or lipid measurements (which, for example, can mediate the effects of adiposity). Similarly, we did not adjust for BMI when analyzing other measures of adiposity (eg, waist circumference). Participants in the UK Biobank were censored at first nonfatal CVD event, death, or study exit, whichever occurred first. Participants in ERFC were censored at death or study exit. Because nonfatal CVD may result in hospitalization (which may, in turn, lead to VTE outcomes), sensitivity analyses additionally censored at the first nonfatal CVD event in ERFC.

To correct for regression dilution caused by variability in levels of continuous risk factors, we regressed serial measurements of risk factors obtained from up to 146 749 participants in ERFC (mean interval, 8.4 years) and up to 24 235 participants in UK Biobank (mean interval, 5.2 years) on baseline levels of the relevant characteristics. Correction for within-person variation in risk factors was achieved by use of conditional expectations of long-term average levels (termed *usual levels*) of the risk factors, which were predicted from regression calibration models and used in estimation of HRs, as described previously.^29^

To characterize shapes of associations, HRs calculated within overall fifths of baseline exposure values were plotted against mean usual values of the relevant risk factor within each fifth. We used the Plummer method to estimate 95% CIs from the variances that corresponded to the amount of information underlying each group (including the reference category).^30^

Because a further aim of the study was to compare associations of risk factors with VTE vs CHD outcomes within the same cohorts, we defined a competing risk model using a record duplication approach, allowing for simultaneous cause-specific hazard regression to estimate cause-specific HRs for each type of event. In ERFC, we stratified the cause-specific regression model by cohort to allow for a different baseline hazard function in each study. We tested for differences in associations with VTE vs CHD based on the interaction between each exposure variable and the event type indicator variable.^31^

Analyses were carried out in Stata version 13 (StataCorp). Because of the number of statistical tests done, principal emphasis was given to findings with a *P *value less than .001, and all *P *values were 2-sided.

## Results

Data were available for 731 728 participants from 75 ERFC cohorts and 421 537 participants from UK Biobank (Table) (eTable 1 in the Supplement). The mean (SD) age at baseline was 51.9 (9.0) years in ERFC and 56.4 (8.1) years in UK Biobank; 403 396 participants (55.1%) in the ERFC and 233 699 (55.4%) in UK Biobank were female. Most participants in ERFC were enrolled in either Europe (369 757 of 731 728 [50.5%]) or North America (315 278 of 731 728 [43.1%]). During a median follow-up of 15.4 years, 1041 fatal VTE events and 25 131 fatal CHD events were recorded in the ERFC. In UK Biobank, 2321 fatal or nonfatal VTE events and 3385 fatal or nonfatal CHD events were recorded during a median follow-up of 6.1 years.

**Table.** Summary of Baseline Characteristics and Outcomes Recorded

Characteristic	ERFC			UK Biobank^a^	
	No. of Cohorts	No.	Measure	No.	Measure
Demographic and lifestyle factors, No. (%)					
Age at baseline survey, mean (SD), y	75	731 728	51.9 (9.0)	421 537	56.4 (8.1)
Male	70	731 728	328 332 (44.9)	421 537	187 838 (44.6)
Current smoker	75	731 728	222 016 (30.3)	421 537	43 847 (10.4)
History of diabetes	74	731 728	25 982 (3.6)	421 537	17 622 (4.2)
Current alcohol drinker	58	386 831	271 499 (70.2)	421 197	389 507 (92.5)
Anthropometric and physical markers, mean (SD)					
Systolic blood pressure, mm Hg	73	566 724	131 (19)	421 179	137 (19)
Diastolic blood pressure, mm Hg	72	565 895	80.0 (10.9)	421 181	82.2 (10.1)
Body mass index^b^	75	731 728	25.4 (4.2)	421 537	27.2 (4.7)
Waist-to-hip ratio	34	264 787	0.85 (0.08)	421 440	0.87 (0.09)
Waist circumference, cm	36	265 465	87.6 (12.5)	421 464	89.6 (13.2)
Lipid-related markers, mean (SD)					
Total cholesterol levels, mg/dL	68	455 177	222.0 (43.6)	NA	NA
Non-HDL cholesterol levels, mg/dL	57	311 888	171.0 (44.8)	NA	NA
HDL cholesterol levels, mg/dL	57	312 207	52.9 (14.7)	NA	NA
Log triglyceride levels, mg/dL^c^	56	322 096	4.79 (0.53)	NA	NA
Apolipoprotein B levels, mg/dL	20	80 712	103 (29)	NA	NA
Apolipoprotein A1 levels, mg/dL	20	84 483	137 (33)	NA	NA
Log Lp(a) levels, mg/dL^d^	18	66 382	2.20 (1.20)	NA	NA
Metabolic and inflammatory markers, mean (SD)					
Fasting glucose levels, mg/dL	33	130 322	88.5 (24.3)	NA	NA
Log CRP levels, mg/L^e^	28	70 855	0.46 (1.07)	NA	NA
Fibrinogen levels, mg/dL	29	115 002	241.2 (68.7)	NA	NA
Albumin levels, g/dL	25	115 309	4.29 (0.39)	NA	NA
Study period, median (5th centile-95th centile)^f^					
Baseline survey year	75	731 728	1986 (1971-2000)	421 537	2009 (2007-2010)
Latest follow-up year	75	731 728	2004 (1989-2011)	421 537	2016 (2016-2016)
Outcomes, No.					
Time to event or censoring, median (5th centile-95th centile), y	75	731 728	15.4 (5.5-32.0)	421 537	6.1 (4.8-7.5)
Total follow up, person-years in millions	75	731 728	12.807	421 537	2.566
Non-fatal MI	NA^g^	NA	NA	421 537	2808
Fatal CHD	75	731 728	25 131	421 537	577
VTE	75	731 728	1041	421 537	2321
Nonfatal VTE	NA^g^	NA	NA	421 537	2234
Fatal VTE	75	731 728	1041	421 537	87
Pulmonary embolism	75	731 728	855	421 537	1273
Deep venous thromboembolism	75	731 728	186	421 537	1048
Unprovoked VTE	NA	NA	NA	421 537	1465
Provoked VTE	NA	NA	NA	421 537	856

Abbreviations: CHD, coronary heart disease; CRP, C-reactive protein; ERFC, Emerging Risk Factors Collaboration; HDL, high-density lipoprotein; Lp(a), lipoprotein(a); MI, myocardial infarction; NA, not applicable; PE, pulmonary embolism; VTE, venous thromboembolism.

SI conversion factor: To convert cholesterol to millimoles per liter, multiply by 0.0259; triglycerides to millimoles per liter, multiply by 0.0113; apolipoprotein to grams per liter, multiply by 0.01; Lp(a) to micromoles per liter, multiply by 0.0357; fasting glucose to micromoles per liter, multiply by 0.0555; CRP to nanomoles per liter, multiply by 9.524; fibrinogen to grams per liter, multiply by 0.01; and albumin to grams per liter, multiply by 10.

^a^ At the time of these analyses, data on plasma biomarkers were not available in UK Biobank.

^b^ Body mass index calculated as weight in kilograms divided by height in meters squared.

^c^ Median (interquartile range) triglyceride level was 117 (82-170) mg/dL.

^d^ Median (interquartile range) Lp(a) level was 9 (4-25) mg/dL.

^e^ Median (interquartile range) CRP level was 1.48 (0.72-3.15) mg/dL.

^f^ Follow-up and outcome summaries among participants with complete data on age, sex, smoking status, history of diabetes, and body mass index.

^g^ Most of the studies in ERFC did not ascertain nonfatal VTE outcomes; hence, analyses in ERFC were restricted to comparison of fatal CHD outcomes only.

**Figure 1.**
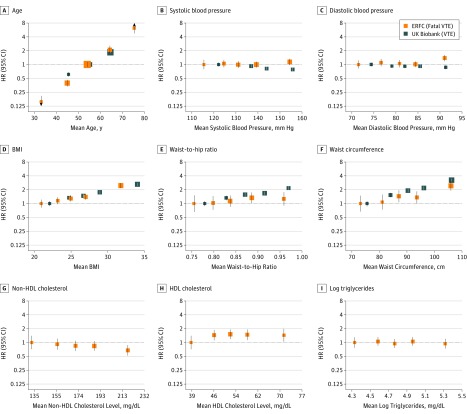
Hazard Ratios (HRs) for Venous Thromboembolism (VTE) by 10-Year Age Groups and Fifths of Continuous Factors All comparisons were adjusted for age, sex, smoking status, history of diabetes, and usual body mass index (BMI; calculated as weight in kilograms divided by height in meters squared) (waist-to-hip ratio and waist circumference were not adjusted for usual BMI). The reference category is age 50 to 59 years for age and is the bottom fifth for all other continuous variables. Associations involve Emerging Risk Factors Collaboration (ERFC) data for fatal VTE and UK Biobank data for VTE. Data on cholesterol and triglyceride levels were unavailable in UK Biobank at the time of analysis. Most UK Biobank participants were aged between 40 and 69 years at baseline. The dotted line indicates the reference value. HDL indicates high-density lipoprotein.

**Figure 2.**
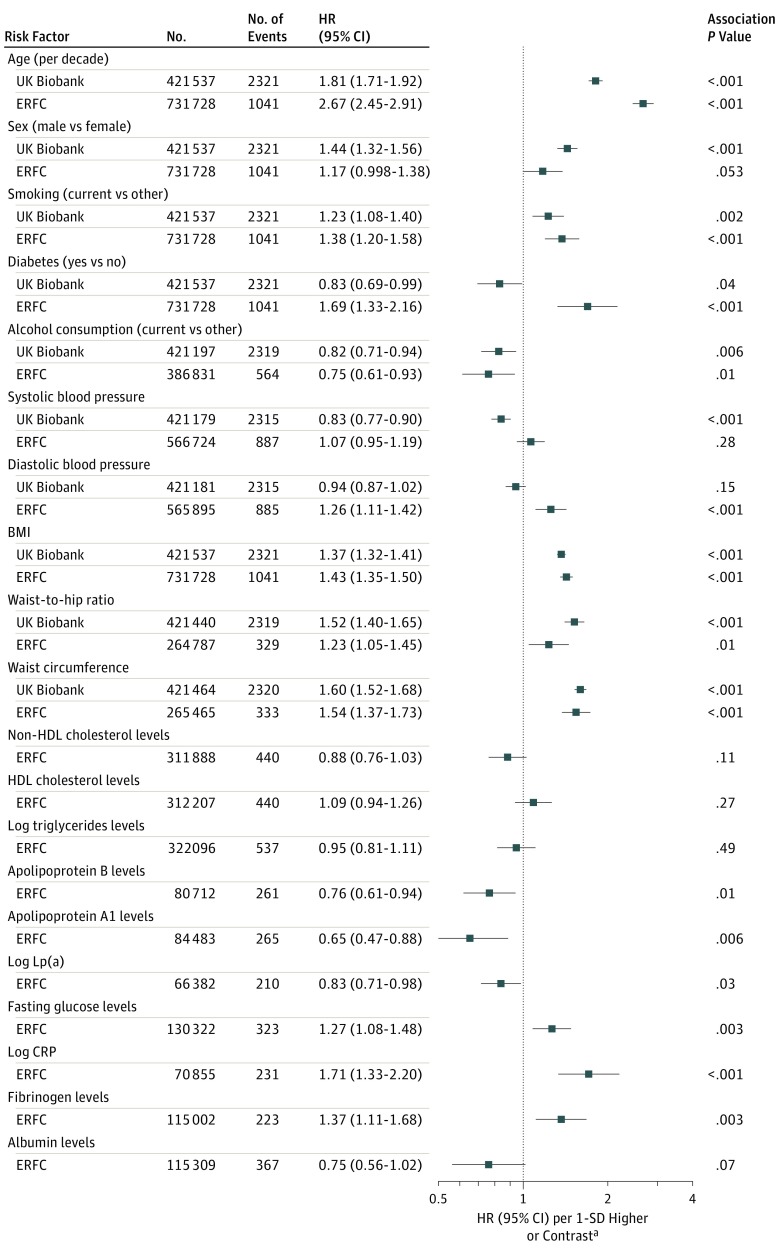
Hazard Ratios (HRs) for Venous Thromboembolism (VTE) for Established Cardiovascular Risk Factors All comparisons were adjusted for age, sex, smoking status, history of diabetes, and usual body mass index (BMI) (waist-to-hip ratio and waist circumference were not adjusted for usual BMI). Associations involve Emerging Risk Factors Collaboration (ERFC) data for fatal VTE and UK Biobank data for VTE. CRP indicates C-reactive protein; HDL, high-density lipoprotein; Lp(a), lipoprotein(a). ^a^Hazard ratios are presented per 1-SD higher usual risk factor level unless otherwise indicated.

Associations of several risk factors with VTE were approximately log-linear (Figure 1). Older age was associated with higher risk of VTE, with an approximately 2.8-fold higher risk per decade in ERFC and 1.8-fold higher risk per decade in UK Biobank (Figure 2). Compared with females, males had a higher risk of VTE in UK Biobank (HR, 1.44; 95% CI, 1.32-1.56), somewhat less so in ERFC (HR, 1.17; 95% CI, 0.998-1.38). Current smoking was associated with VTE risk in ERFC (HR, 1.38; 95% CI, 1.20-1.58), but somewhat less so in UK Biobank (HR, 1.23; 95% CI, 1.08-1.40). Markers of adiposity (BMI, waist-to-hip ratio, and waist circumference) were positively associated with higher VTE risk in both ERFC and UK Biobank. For example, HRs per 1-SD higher BMI were 1.43 (95% CI, 1.35-1.50) in ERFC and 1.37 (95% CI, 1.32-1.41) in UK Biobank. Current alcohol consumption was inversely associated with VTE risk in both ERFC (HR, 0.75; 95% CI, 0.61-0.93) and UK Biobank (HR, 0.82; 95% CI, 0.71-0.94). In exploratory analyses restricted to current drinkers in UK Biobank (which should limit the effects of certain residual biases, such as reverse causality related to sick quitters^32^), we found that the inverse association between amount of alcohol consumed and VTE risk persisted (eFigure 1 in the Supplement).

By contrast, for some other risk factors we studied, we noted potentially directionally discordant associations across ERFC and UK Biobank. For example, 1-SD higher systolic blood pressure was not associated with risk of VTE in ERFC (HR, 1.07; 95% CI, 0.95-1.19) but was inversely associated with risk of VTE in UK Biobank (HR, 0.83; 95% CI, 0.77-0.90). Conversely, 1-SD higher diastolic blood pressure was associated with higher risk of VTE in ERFC (HR, 1.26; 95% CI, 1.11-1.42) but was not associated with risk of VTE in UK Biobank (HR, 0.94; 95% CI, 0.87-1.02). In ERFC, history of diabetes was associated with higher risk of VTE (HR, 1.69; 95% CI, 1.33-2.16) as was 1-SD higher fasting baseline glucose concentration (HR, 1.27; 95% CI, 1.08-1.48), while in UK Biobank, history of diabetes was inversely associated with risk of VTE (HR, 0.83; 95% CI, 0.69-0.99). To investigate whether these discordant associations chiefly reflected the different VTE outcomes recorded across ERFC and UK Biobank, we restricted analysis to the UK Biobank (which had recorded both fatal and nonfatal VTE outcomes). In UK Biobank–specific analyses, we found a similar pattern of difference of HRs for fatal vs nonfatal VTEs with blood pressure and diabetes to that observed in our comparison across ERFC and UK Biobank (eFigure 2 in the Supplement). This result suggests that blood pressure and diabetes may have differing associations with fatal vs nonfatal VTEs.

At the time of our analysis, data on plasma biomarkers were available in the ERFC but not in UK Biobank (Figure 2). In the ERFC, apolipoprotein B, apolipoprotein A, and lipoprotein(a) levels each showed suggestively inverse associations with risk of VTE, whereas triglyceride, non–high-density lipoprotein cholesterol, and high-density lipoprotein cholesterol levels each showed no associations. Fasting glucose, C-reactive protein, and fibrinogen levels were each associated with higher risk of VTE.

**Figure 3.**
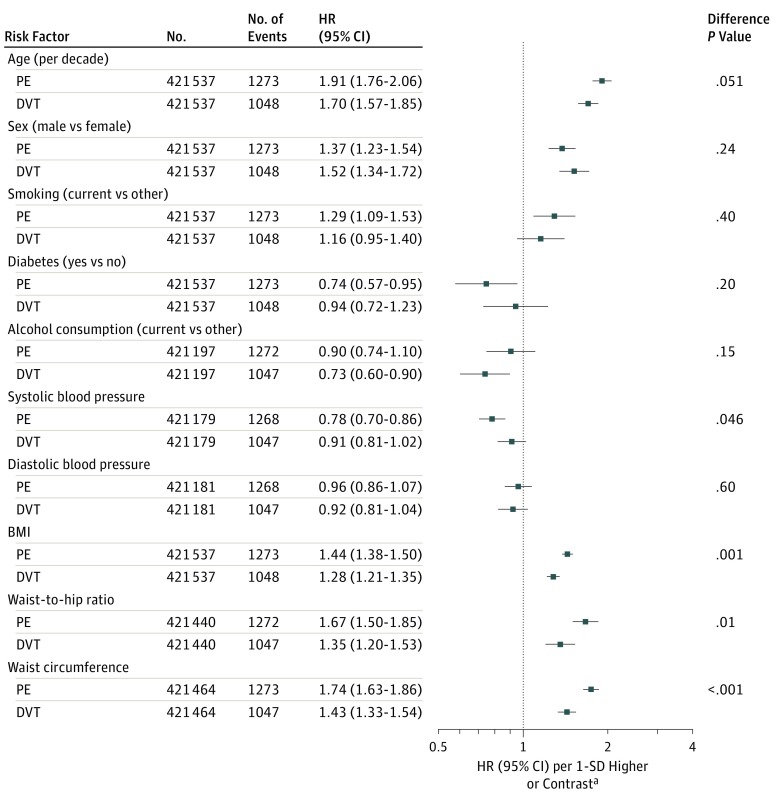
Hazard Ratios (HRs) for Pulmonary Embolism (PE) vs Deep Vein Thrombosis (DVT) for Established Cardiovascular Risk Factors in UK Biobank All comparisons were adjusted for age, sex, smoking status, history of diabetes, and usual body mass index (BMI) (waist-to-hip ratio and waist circumference were not adjusted for usual BMI). Associations involve UK Biobank data only. ^a^Hazard ratios are presented per 1-SD higher usual risk factor level unless otherwise indicated.

**Figure 4.**
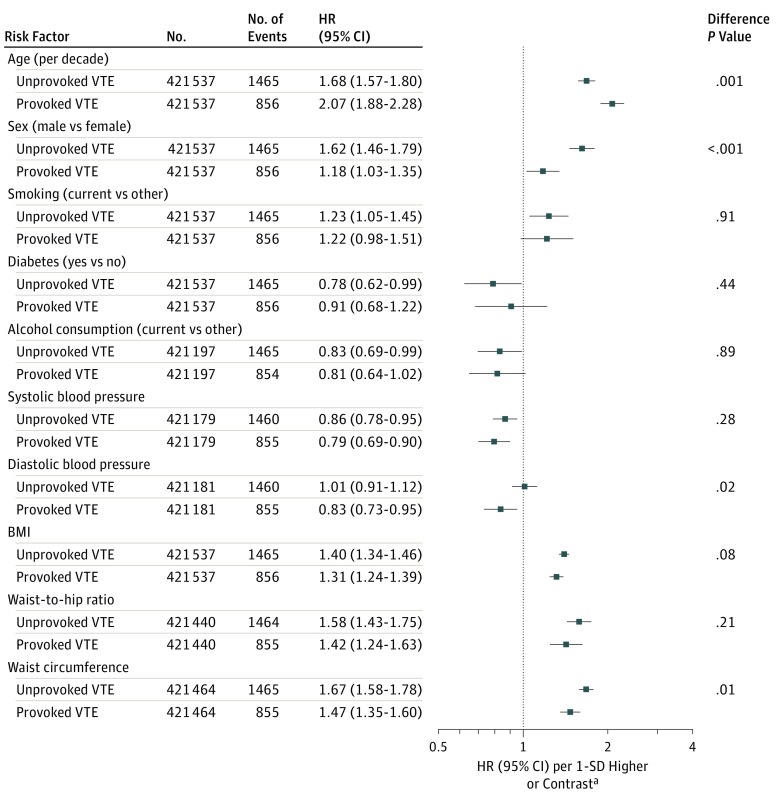
Hazard Ratios (HRs) for Unprovoked vs Provoked Venous Thromboembolism (VTE) for Established Cardiovascular Risk Factors in UK Biobank All comparisons were adjusted for age, sex, smoking status, history of diabetes, and usual body mass index (BMI) (waist-to-hip ratio and waist circumference were not adjusted for usual BMI). Associations involve UK Biobank data only. ^a^Hazard ratios are presented per 1-SD higher usual risk factor level unless otherwise indicated.

In analyses comparing PE with DVT, higher BMI and higher waist circumference had stronger associations with PE than DVT (Figure 3). Further analyses that subcategorized VTE outcomes as provoked vs unprovoked in UK Biobank did not reveal major differences in the associations of most CVD risk factors, with the exceptions of older age and male sex (Figure 4).

In analyses comparing VTE with CHD outcomes, associations were stronger for CHD in both ERFC and UK Biobank for most risk factors, including age, male sex, current smoking status, history of diabetes, higher systolic and diastolic blood pressure, and proatherogenic lipid levels (eFigures 3 and 4 in the Supplement). In contrast, higher BMI and waist circumference had somewhat stronger associations with VTE compared with CHD, whereas circulating inflammatory markers were associated with both conditions to a broadly similar extent (eFigures 3 and 4 in the Supplement). Findings were broadly similar in sensitivity analyses that did not adjust for BMI (eTable 2 in the Supplement), excluded participants with history of cancer diagnosis at baseline (eFigure 5 in the Supplement), censored for first CVD events in ERFC (eFigure 6 in the Supplement), and used baseline levels of risk factors, except for the expected decrease in the magnitudes of association when not correcting for within-person variability in the continuous variables (eFigures 7-9 in the Supplement).

## Discussion

In this analysis of individual-level data on several established cardiovascular risk factors from more than 1.1 million participants in 76 cohorts, we found that older age, smoking, and higher levels of adiposity were clearly associated with higher risk of VTE. These findings suggest that there is overlap in at least some major population determinants of important venous and arterial thrombotic diseases.

Our study characterized dose-response associations between several clinical measures of adiposity (eg, waist circumference and BMI) and VTE risk and showed no evidence of a threshold below which leaner body habitus stopped being associated with lower VTE risk. The association of obesity with VTE is supported by previous mendelian randomization studies of genetic variants associated with increased adiposity, which are also associated with increased risk of VTE.^33,34^ Furthermore, we found that associations of BMI and waist circumference were somewhat stronger with PE vs DVT and about twice as strong with VTE vs CHD. These data suggest that efforts to combat the entire spectrum of obesity and overweight should yield important benefits for VTE prevention.

As regards risk behaviors, our study confirmed the known association of current smoking with risk of VTE.^9,13^ This association was similar in magnitude for PE and DVT outcomes but weaker than that observed for CHD. Previous studies have suggested that much of the excess risk of VTE in smokers was because of increased hospitalization for smoking-related diseases, including cancer.^35,36^ However, in our analysis, smoking was similarly associated with both provoked and unprovoked VTE; furthermore, HRs did not change appreciably after exclusion of participants with history of cancer diagnosis at baseline. We also noted a pattern of association between alcohol consumption and VTE similar to that reported in previous studies of alcohol consumption and nonfatal myocardial infarction.^32,37,38^ (By contrast, alcohol consumption has previously been positively associated with risks of fatal coronary disease, stroke, and heart failure.) Although previous studies have reported that moderate alcohol consumption is associated with lower levels of hemostatic factors (eg, fibrinogen, factor VII, and von Willebrand factor),^39,40^ further studies are needed to determine whether moderate alcohol consumption has a causal role in VTE.

Our study identified potentially inverse associations of proatherogenic lipid levels with VTE. For example, apolipoprotein B and lipoprotein(a) levels were each associated with lower risk of VTE, a finding that awaits further elucidation.^41^ Proinflammatory soluble biomarkers (eg, C-reactive protein) were positively associated with VTE, a finding consistent with the associations we observed for CHD outcomes. Although previous mendelian randomization studies suggest that CRP and fibrinogen levels are unlikely to be direct causal factors in CHD,^42,43^ such genetic epidemiological data are sparser in relation to VTE.

It is not clear why our study found inconsistent associations of blood pressure and history of diabetes with VTE outcomes across UK Biobank and the ERFC. One potential explanation is that these data sources recorded mostly differing types of VTE outcomes, ie, UK Biobank involved mostly nonfatal outcomes whereas ERFC involved only fatal outcomes. Our exploratory analysis of UK Biobank data was consistent with this explanation, as it found differing results with blood pressure and diabetes for fatal VTE vs nonfatal VTE similar to those observed in comparisons across UK Biobank and the ERFC. However, future studies with more detailed clinical information will be needed to understand these possible differences with greater confidence.

### Strengths and Limitations

Our study had major strengths. It avoided the limitations of retrospective case-control study designs by analyzing prospective cohort data on more than 1.1 million participants without CVD at baseline. Access to individual participant data avoided the limitations of literature-based meta-analyses. It also enabled a common approach to adjustment for potential confounding factors, time-to-event analyses, correction for regression dilution bias, and head-to-head comparisons of VTE and CHD. We explored idiopathic VTE vs VTE provoked by established risk factors (such as cancer or prolonged immobility), albeit using pragmatic record-based definitions.^44^ The generalizability of our results was enhanced by inclusion of data from 75 prospective studies in ERFC recruited from 1960 through 2008 in 18 different countries. To enhance power and evaluate the relevance of findings to the contemporary situation, we included data from UK Biobank, which recruited participants from 2006 to 2010.

Our study also had limitations. We did not routinely have information in ERFC data on non-CVD risk factors for VTE (eg, oral contraception use) or medication use (eg, anticoagulants). Misclassification of disease outcomes could have arisen from inaccuracies in hospital discharge records and death certificates, diluting the strength of the observed associations.^45,46,47^ However, 2 observations argue against major disease misclassification in our study. First, we observed associations of measures of adiposity with VTE risk similar in size to those previously reported in much smaller studies based on detailed validation of VTE events.^6^ Second, we observed directionally opposite associations of proatherogenic lipid levels with VTE and CHD outcomes despite the 2 conditions having similar clinical presentations.

## Conclusions

Among a panel of several established cardiovascular risk factors, older age, smoking, and adiposity were consistently associated with higher VTE risk. There is overlap in at least some of the major population determinants of important venous and arterial thrombotic diseases.

## Notes

Supplement. **eAppendix.** Outcome definitions. **eTable 1.** Cohort-specific summaries of selected characteristics and recorded outcomes. **eTable 2.** Hazard ratios for VTE per 1-SD higher usual risk factor value or group contrast, with and without adjustment for BMI. **eFigure 1.** Hazard ratios for fatal/nonfatal VTE by alcohol consumption among current drinkers in UK Biobank. **eFigure 2.** Hazard ratios for nonfatal vs fatal VTE per 1-SD higher usual risk factor value or group contrast, with and without adjustment for BMI. **eFigure 3.** Hazard ratios for fatal CHD vs fatal VTE (left) and fatal/nonfatal CHD vs VTE (right) per 1-SD higher usual risk factor value or group contrast. **eFigure 4.** Hazard ratios for fatal CHD vs fatal VTE per 1-SD higher usual levels of biochemical markers. **eFigure 5.** Hazard ratios for unprovoked and provoked VTE per 1-SD higher usual risk factor value or group contrast in UK Biobank after excluding participants with history of cancer. **eFigure 6.** Hazard ratios for fatal CHD vs fatal VTE per 1-SD higher usual risk factor value or group contrast, with censoring at first event. **eFigure 7.** Hazard ratios for VTE per 1-SD higher baseline risk factor value or group contrast. **eFigure 8.** Hazard ratios for fatal CHD vs fatal VTE (left) and fatal/nonfatal CHD vs VTE (right) per 1-SD higher baseline risk factor value or group contrast. **eFigure 9.** Hazard ratios for fatal CHD vs fatal VTE per 1-SD higher baseline levels of biochemical markers.
