# Supplementary material for: Cardiovascular Risk Factors Associated With Venous Thromboembolism
Source: JAMA Cardiol. 2019 Jan 16;4(2):43–53. doi: 10.1001/jamacardio.2018.4537 (PMC6386140; doi:10.1001/jamacardio.2018.4537)
Supplement: Supplement. — eAppendix. Outcome definitions. eTable 1. Cohort-specific summaries of selected characteristics and recorded outcomes. eTable 2. Hazard ratios for VTE per 1-SD higher usual risk factor value or group contrast, with and without adjustment for BMI. eFigure 1. Hazard ratios for fatal/nonfatal VTE by alcohol consumption among current drinkers in UK Biobank. eFigure 2. Hazard ratios for nonfatal vs fatal VTE per 1-SD higher usual risk factor value or group contrast, with and without adjustment for BMI. eFigure 3. Hazard ratios for fatal CHD vs fatal VTE (left) and fatal/nonfatal CHD vs VTE (right) per 1-SD higher usual risk factor value or group contrast. eFigure 4. Hazard ratios for fatal CHD vs fatal VTE per 1-SD higher usual levels of biochemical markers. eFigure 5. Hazard ratios for unprovoked and provoked VTE per 1-SD higher usual risk factor value or group contrast in UK Biobank after excluding participants with history of cancer. eFigure 6. Hazard ratios for fatal CHD vs fatal VTE per 1-SD higher usual risk factor value or group contrast, with censoring at first event. eFigure 7. Hazard ratios for VTE per 1-SD higher baseline risk factor value or group contrast. eFigure 8. Hazard ratios for fatal CHD vs fatal VTE (left) and fatal/nonfatal CHD vs VTE (right) per 1-SD higher baseline risk factor value or group contrast. eFigure 9. Hazard ratios for fatal CHD vs fatal VTE per 1-SD higher baseline levels of biochemical markers. [file jamacardiol-4-43-s001.pdf]

Supplementary Online Content

Gregson J, Kaptoge S, Bolton T, et al; Emerging Risk Factors Collaboration. Cardiovascular risk factors associated with venous thromboembolism. *JAMA Cardiol*. Published online January 16, 2019. doi:10.1001/jamacardio.2018.4537

eAppendix. Outcome definitions

eTable 1. Cohort-specific summaries of selected characteristics and recorded outcomes

eTable 2. Hazard ratios for VTE per 1-SD higher usual risk factor value or group contrast, with and without adjustment for BMI

eFigure 1. Hazard ratios for fatal/nonfatal VTE by alcohol consumption among current drinkers in UK Biobank

eFigure 2. Hazard ratios for nonfatal vs fatal VTE per 1-SD higher usual risk factor value or group contrast, with and without adjustment for BMI

eFigure 3. Hazard ratios for fatal CHD vs fatal VTE (left) and fatal/nonfatal CHD vs VTE (right) per 1-SD higher usual risk factor value or group contrast

eFigure 4. Hazard ratios for fatal CHD vs fatal VTE per 1-SD higher usual levels of biochemical markers

eFigure 5. Hazard ratios for unprovoked and provoked VTE per 1-SD higher usual risk factor value or group contrast in UK Biobank after excluding participants with history of cancer

eFigure 6. Hazard ratios for fatal CHD vs fatal VTE per 1-SD higher usual risk factor value or group contrast, with censoring at first event

eFigure 7. Hazard ratios for VTE per 1-SD higher baseline risk factor value or group contrast

eFigure 8. Hazard ratios for fatal CHD vs fatal VTE (left) and fatal/nonfatal CHD vs VTE (right) per 1-SD higher baseline risk factor value or group contrast

eFigure 9. Hazard ratios for fatal CHD vs fatal VTE per 1-SD higher baseline levels of biochemical markers

This supplementary material has been provided by the authors to give readers additional information about their work.

eAppendix. Outcome definitions

Definition of venous thromboembolism (VTE) and coronary heart disease (CHD)
In both ERFC and UKBB, VTE and CHD outcomes were defined based on International Classification of Disease (ICD) codes (**eAppendix Table 1**).

eAppendix Table 1. ICD codes for VTE and CHD outcomes

Outcome \ ICD version	ICD-10	ICD-9	ICD-8
Venous thromboembolism (VTE)			
Pulmonary embolism (PE)	I26	415	450
Deep venous thromboembolism (DVT)	I80-I82	451-453	451-453
Coronary heart disease (CHD)			
Non-fatal MI	I21-I23	410,412	410,412
CHD death	I24-I25	411,414	411,414

Definition of provoked venous thromboembolism in UKBB
For UK Biobank participants, it was possible to use available health-related outcome data to subcategorise the VTE as provoked or unprovoked.

According to the NICE Guideline Development Group (GDG), a provoked VTE is defined as a VTE that occurred in the presence of an antecedent (within 3 months) and transient major clinical risk factor for VTE (for example surgery, trauma or significant immobility).[1] The NICE GDG also consider VTE that occurs in association with hormonal therapy (oral contraceptive or hormone replacement therapy) to be provoked because it has been shown that people having these therapies have a lower risk of VTE recurrence.[2] Definitions of provoked VTE have also been extended to include active cancer, pregnancy or puerperium, long-distance travel, active infectious disease, acute myocardial infarction, paresis/paralysis of leg and heart failure.[1]

Our definition of provoked VTE was pragmatic and reflective of the health-related outcome data available in UK Biobank, which includes cancer registry data, containing coded data on cancer incidence obtained through linkage to national cancer registries, and hospital in-patient data, containing data on hospital admissions obtained through linkage to external data providers (in-patients are defined as persons who are admitted to hospital and occupy a hospital bed).

We defined a VTE as provoked if, in the 90-day period preceding the VTE, the participant was recorded as having: a malignant neoplasm (as per cancer registry data); or started/ended a hospital episode with a main diagnosis code relating to malignant neoplasm, heart failure, infectious disease or trauma; or a hospital episode that included certain types of operation. The specific International Statistical Classification of Diseases and Related Health Problems (ICD)[3] codes and Office of Population Censuses and Surveys (OPCS) Classification of Interventions and Procedures version 4[4] chapters that are included in our definition have been summarised in **eAppendix Table 2**. A VTE not defined as provoked was defined as unprovoked.

eAppendix Table 2. Antecedent events used to define provoked VTE in UKBB.

Event	Source	ICD-10	OPCS-4
Malignant neoplasm	Cancer register, Hospital in-patient	C00-C97	
Heart failure	Hospital in-patient	I50	
Infectious, bacterial, viral and parasitic diseases (except hepatitis)	Hospital in-patient	A00-A99, B00-B14, B20-B99	
Trauma	Hospital in-patient	S00-S99, T00-T35, T79-T98, V01-V99, W00-W19, W50-W64	
Operation	Hospital in-patient		Chapters: A, B, E, G, H, J, K, L, M, N, O, P, Q, R, T, V, W, X, Y, Z

© 2019 Gregson J et al. *JAMA Cardiol.*

Exclusions

The initial UK biobank dataset comprised 502,601 participants who hadn't withdrawn by September 2018. We excluded 76,857 (15.3%) participants because of history of CVD/VTE at baseline (including 13638 with history of VTE) and a further 4207 (0.8%) because of incomplete data on smoking status, diabetes status, and BMI; leaving 421,537 (83.9%) participants included in analysis.

List of study acronyms*

Abbreviation	Full name
BRHS	British Regional Heart Study
BRUN	Bruneck Study
BWHHS	British Women's Heart and Health Study
CAPS	Caerphilly Prospective Study
CASTEL	Cardiovascular Study in the Elderly
COPEN	Copenhagen City Heart Study
DRECE	Diet and Risk of Cardiovascular Disease in Spain
EAS	Edinburgh Artery Study
ESTHER	Epidemiologische Studie zu Chancen der Verhutung und optimierten Therapie chronischer Erkrankungen in der alteren Bevolkerung
FINE_IT	Finland, Italy and Netherlands Elderly Study
FINNMARK	Cohort of Norway
FINRISK92	Finrisk Cohort 1992
FINRISK97	Finrisk Cohort 1997
GOTO33	Goteborg Study 1933
GOTOW	Population Study of Women in Göteborg, Sweden
GRIPS	Göttingen Risk Incidence and Prevalence Study
HCS	Hertfordshire Cohort Study
HOORN	Hoorn Study
HUBRO	Cohort of Norway
KARELIA	North Karelia Project
KIHD	Kuopio Ischaemic Heart Disease Study
LEADER	Lower Extremity Arterial Disease Event Reduction Trial
MATISS83	Progetto CUORE
MCVDRFP	Monitoring of CVD Risk Factors Project
MDC	Malmö Diert and Cancer Cardiovascular Study
MICOL	Risk Factors and Life Expectancy Pooling Project
MONFRI86	Progetto CUORE
MONFRI89	Progetto CUORE
MONICA_KORA1	MONICA/KORA Augsburg Survey S1
MONICA_KORA2	MONICA/KORA Augsburg Survey S2
MONICA_KORA3	MONICA/KORA Augsburg Survey S3
MORGEN	Dutch Monitoring Project on Risk Factors for Chronic Diseases
MOSWEGOT	MONICA Göteborg Study
MPP	Malmö Preventive Project
MRCOLD	MRC Study of Older People
NFR	Risk Factors and Life Expectancy Pooling Project
NPHSII	Northwick Park Heart Study II
OPPHED	Cohort of Norway
OSLO	Oslo Study
OSLO2	Cohort of Norway
PREVEND	Prevention of Renal and Vascular End Stage Disease Study
PROCAM	Prospective Cardiovascular Münster Study
ProspectEPIC	Prospect-EPIC Utrecht
REYK	Reykjavik Study
RS_I	The Rotterdam Study
SHHEC	Scottish Heart Health Extended Cohort
SHIP	Study of Health in Pomerani
SPEED	Speedwell Study
TROMSØ	Tromsø Study
ULSAM	Uppsala Longitudinal Study of Adult Men
WCWC	Wuertemberg Construction Workers Cohort
WHITEI	Whitehall I Study
WHITEII	Whitehall II Study
ZUTE	Zutphen Elderly Study
ARIC	Atherosclerosis Risk in Communities Study
CHA	Chicago Heart Association Detection Project In Industry
CHARL	Charleston Heart Study
HONOL	Honolulu Heart Program
HPFS1	Health Professionals Follow-up Study
MRFIT	Multiple Risk Factor Intervention Trial 1
NHANESI	National Health and Nutrition Examination Survey I
NHS1	Nurses' Health Study
QUEBEC	Quebec Cardiovascular Study
RANCHO	Rancho Bernardo Study
SHS	Strong Heart Study
WHIOS	Women's Health Initiative (Oberservational Study)
AUSDIAB	Australian Diabetes Study
BHS	Busselton Health Study

© 2019 Gregson J et al. *JAMA Cardiol.*

DUBBO	Dubbo Study of the Elderly
GOH	The Glucose Intolerance, Obesity and Hypertension Study
HIMS	Health in Men Study
HISAYAMA	Hisayama Study
IKNS	Ikawa, Kyowa, and Noichi Study
PRHHP	Puerto Rico Heart Health Program
TARFS	Turkish Adult Risk Factor Study
ERFC	Emerging Risk Factors Collaboration
UKBB	UK Biobank

* The cohorts are grouped according to 3 geographic locations: Western Europe, North America, and Other.

eReferences

1. National Institute for Health and Care Excellence. Venous thromboembolic diseases : diagnosis , management and thrombophilia testing. *NICE Guidel.* 12–15 (2015). doi:10.1056/NEJMoa023153
2. Barnes, G. D., Kanthi, Y. & Froehlich, J. B. Venous thromboembolism: Predicting recurrence and the need for extended anticoagulation. *Vasc. Med.* **20,** 143–52 (2015).
3. WHO. *International Statistical Classification of Diseases and Related Health Problems (International Classification of Diseases)(ICD) 10th Revision - Version:2010*. *Occupational Health* **1,** (2010).
4. NHS Digital. *National Clinical Coding Standards - OPCS-4 (2017) Reference Book*. (TSO (The Stationery Office), 2017).

© 2019 Gregson J et al. *JAMA Cardiol.*

eTable 1. Cohort-specific summaries of selected characteristics and recorded outcomes*

											Number of first-recorded CHD or VTE events**							Number of fatal CHD or VTE events**				
Cohort abbreviation	Country	Median year of baseline	Maximum year of follow up[$]	Total subjects	Age at survey (yrs) mean (sd)	Sex Male (%)	Smoking Current (%)	Diabetes Yes (%)	BMI (kg/m^2) mean (sd)	Years of follow up to first event or censoring, Median (5th, 95th percentiles)	Nonfatal MI and Fatal CHD	Nonfatal MI	Venous thromboembolism (VTE)	Nonfatal VTE[†]	Pulmonary embolism (PE)	Deep venous thrombosis (DVT)	Person-years of first event	Fatal CHD	Fatal VTE	Fatal PE	Fatal DVT	Person-years of fatal event
BRHS	UK	1979	2005	6808	50 (6)	6808 (100)	3643 (54)	77 (1.1)	25 (3)	24.5 (4.7 to 25.4)	1214	675	22	0	12	10	137100	747	26	15	11	146605
BRUN	Italy	1990	2010	794	57 (11)	387 (49)	193 (24)	28 (3.5)	25 (4)	20.2 (4.4 to 20.5)	63	28	6	0	6	0	13479	43	6	6	0	13911
BWHHS	UK	2000	2014	2764	68 (5)	0 (0)	317 (11)	109 (3.9)	27 (5)	12.2 (3.5 to 13.3)	107	52	7	0	4	3	30667	81	8	4	4	35946
CAPS	UK	1981	2000	2123	52 (5)	2123 (100)	1176 (55)	28 (1.3)	26 (4)	13.0 (4.0 to 13.0)	251	139	7	0	7	0	24950	145	7	7	0	25982
CASTEL	Italy	1984	1996	2536	73 (5)	965 (38)	330 (13)	326 (12.9)	26 (4)	11.2 (2.4 to 14.0)	115	20	29	0	29	0	24963	96	29	29	0	25376
COPEN	Denmark	1993	2013	8319	57 (15)	3577 (43)	4059 (49)	233 (2.8)	25 (4)	16.6 (2.7 to 18.5)	625	566	35	0	32	3	115099	253	43	39	4	133742
DRECE	Spain	1991	2011	2852	39 (11)	1377 (48)	1164 (41)	162 (5.7)	26 (5)	19.4 (17.1 to 19.6)	18	0	1	0	1	0	53646	18	1	1	0	53646
EAS	Scotland	1988	2008	1005	64 (6)	503 (50)	214 (21)	43 (4.3)	25 (4)	20.2 (2.8 to 21.3)	93	39	4	0	2	2	15945	72	4	2	2	16704
ESTHER	Germany	2001	2015	7568	61 (7)	3239 (43)	1321 (17)	895 (11.8)	28 (4)	5.0 (1.6 to 5.9)	77	75	0	0	0	0	34841	183	8	6	2	102332
FINE_IT	Italy	1985	2006	458	72 (4)	458 (100)	123 (27)	37 (8.1)	26 (4)	9.8 (1.9 to 21.4)	66	18	1	0	0	1	5023	56	1	0	1	5503
FINNMARK	Norway	2002	2009	5217	59 (10)	2345 (45)	1923 (37)	207 (4.0)	28 (5)	7.5 (5.0 to 7.5)	48	0	1	0	1	0	37780	48	1	1	0	37780
FINRISK92	Finland	1992	2008	5746	44 (11)	2654 (46)	1750 (30)	104 (1.8)	26 (4)	16.8 (4.4 to 16.9)	138	103	6	0	6	0	87854	48	6	6	0	93977
FINRISK97	Finland	1997	2008	7163	49 (12)	3512 (49)	1866 (26)	226 (3.2)	27 (4)	11.8 (3.1 to 11.9)	112	81	9	0	9	0	77824	39	11	11	0	82955
GOTO33	Sweden	1984	1998	720	51 (0)	720 (100)	264 (37)	25 (3.5)	26 (3)	12.8 (5.7 to 13.1)	27	14	2	0	2	0	8720	13	2	2	0	8868
GOTOW	Sweden	1969	2009	1387	47 (6)	0 (0)	568 (41)	14 (1.0)	24 (4)	32.2 (10.6 to 32.8)	138	90	0	0	0	0	38576	140	1	1	0	46227
GRIPS	Germany	1982	1992	5785	48 (5)	5785 (100)	2179 (38)	113 (2.0)	26 (3)	9.8 (4.8 to 10.0)	299	299	13	0	13	0	53704	70	17	17	0	55362
HCS	UK	2001	2012	2524	66 (3)	1276 (51)	326 (13)	337 (13.4)	27 (4)	8.9 (6.0 to 11.6)	26	0	5	0	2	3	22322	26	5	2	3	22322
HOORN	Netherlands	1991	2005	2219	61 (7)	982 (44)	705 (32)	209 (9.4)	27 (4)	8.8 (3.5 to 9.9)	73	60	3	0	3	0	18406	27	4	4	0	27508
HUBRO	Norway	2001	2009	15290	51 (14)	6699 (44)	4307 (28)	389 (2.5)	26 (4)	8.5 (7.0 to 9.5)	75	0	5	0	3	2	132705	75	5	3	2	132705
KARELIA	Finland	1972	2008	128	46 (10)	65 (51)	35 (27)	128 (100.0)	28 (5)	18.4 (2.8 to 36.8)	45	31	1	0	1	0	2525	30	1	1	0	2920
KIHD	Finland	1987	2009	2063	53 (5)	2063 (100)	632 (31)	92 (4.5)	27 (4)	20.9 (2.9 to 25.1)	404	387	2	0	2	0	36816	155	3	3	0	41296
LEADER	UK	1997	2001	927	68 (9)	927 (100)	382 (41)	149 (16.1)	26 (4)	4.2 (0.9 to 6.2)	99	36	1	0	1	0	3699	66	2	2	0	3890
MATISS83	Italy	1984	2002	2539	51 (10)	1190 (47)	753 (30)	122 (4.8)	28 (4)	18.7 (6.9 to 19.5)	82	46	1	0	1	0	43332	42	1	1	0	43505
MCVDRFP	Netherlands	1989	2007	23506	42 (10)	10937 (47)	10316 (44)	237 (1.0)	25 (4)	16.7 (10.5 to 18.9)	196	0	11	0	8	3	378872	196	11	8	3	378872
MDC	Sweden	1993	2010	5565	57 (6)	2256 (41)	1546 (28)	228 (4.1)	26 (4)	17.7 (5.0 to 19.1)	387	361	4	0	4	0	88885	151	6	6	0	92872
MICOL	Italy	1986	1993	18848	51 (10)	10642 (56)	6158 (33)	939 (5.0)	26 (4)	5.9 (4.5 to 7.1)	102	0	3	0	3	0	112955	102	3	3	0	112955
MONFRI86	Italy	1986	2002	1121	49 (9)	546 (49)	353 (31)	11 (1.0)	27 (4)	16.7 (8.2 to 16.9)	23	17	1	0	1	0	17670	6	1	1	0	17584
MONFRI89	Italy	1989	2002	1094	48 (8)	541 (49)	310 (28)	13 (1.2)	27 (4)	13.6 (7.8 to 13.7)	19	15	1	0	1	0	14230	4	1	1	0	14288
MONICA_KORA1	Germany	1985	1998	871	54 (6)	871 (100)	294 (34)	37 (4.2)	28 (3)	13.0 (3.6 to 13.4)	79	47	5	0	5	0	10107	42	5	5	0	10572
MONICA_KORA2	Germany	1990	1998	3962	53 (12)	1949 (49)	969 (24)	196 (4.9)	27 (4)	7.9 (2.3 to 8.4)	104	63	4	0	4	0	28548	69	7	7	0	30580
MONICA_KORA3	Germany	1995	2009	4490	50 (14)	2198 (49)	1129 (25)	199 (4.4)	27 (5)	14.0 (4.5 to 14.7)	203	133	6	0	6	0	56908	89	12	12	0	59342
MORGEN	Netherlands	1995	2007	18242	46 (9)	8302 (46)	6820 (37)	242 (1.3)	26 (4)	10.8 (8.3 to 13.0)	77	0	4	0	3	1	191042	77	4	3	1	191042
MOSWEGOT	Sweden	1990	2003	4144	47 (11)	1964 (47)	1213 (29)	97 (2.3)	25 (4)	13.9 (7.7 to 19.6)	154	115	7	0	7	0	56279	56	7	7	0	53695
MPP	Sweden	1980	2010	31575	45 (7)	21669 (69)	14265 (45)	1041 (3.3)	25 (4)	27.2 (7.6 to 33.9)	4092	3644	53	0	35	18	797250	2029	82	55	27	841759
MRCOLD	UK	1996	2007	10137	80 (4)	3825 (38)	1222 (12)	695 (6.9)	26 (4)	8.7 (1.2 to 11.7)	1146	0	73	0	47	26	76923	1146	73	47	26	76923
NFR	Italy	1980	1991	3075	55 (5)	3075 (100)	1564 (51)	198 (6.4)	26 (3)	10.2 (6.1 to 11.2)	90	0	1	0	1	0	30183	90	1	1	0	30183
NPHSII	UK	1991	2005	2959	57 (3)	2959 (100)	1100 (37)	72 (2.4)	26 (4)	8.3 (3.4 to 10.4)	192	173	2	1	2	0	22832	60	3	2	1	24054
OPPHED	Norway	2001	2009	9200	52 (12)	4194 (46)	2888 (31)	227 (2.5)	27 (4)	8.5 (7.0 to 9.5)	69	0	2	0	1	1	77693	69	2	1	1	77693
OSLO	Norway	1973	2003	17247	44 (6)	17247 (100)	9596 (56)	148 (0.9)	25 (3)	29.5 (10.9 to 30.5)	1604	0	28	0	15	13	454876	1604	28	15	13	454876
OSLO2	Norway	2000	2009	5025	69 (7)	5025 (100)	1095 (22)	263 (5.2)	26 (3)	9.5 (3.0 to 9.5)	97	0	3	0	1	2	43211	97	3	1	2	43211
PREVEND	Netherlands	1998	2010	7358	49 (12)	3589 (49)	2520 (34)	259 (3.5)	26 (4)	10.6 (4.5 to 11.2)	199	168	3	0	3	0	73179	54	3	3	0	90357
PROCAM	Germany	1983	2003	20151	44 (10)	14582 (72)	7872 (39)	410 (2.0)	26 (4)	10.0 (3.8 to 18.9)	486	367	13	0	13	0	239003	135	13	13	0	239843
ProspectEPIC	Netherlands	1995	2012	15692	57 (6)	0 (0)	3559 (23)	318 (2.0)	26 (4)	14.3 (7.7 to 17.2)	272	195	7	0	5	2	216679	86	7	5	2	219771

© 2019 Gregson J et al. *JAMA Cardiol.*

REYK	Iceland	1974	2008	16756	52 (9)	8028 (48)	7950 (47)	314 (1.9)	25 (4)	24.7 (6.2 to 37.1)	3250	2030	84	0	78	6	401228	2226	100	90	10	432609
RS_I	Netherlands	1992	2005	3825	68 (8)	1425 (37)	890 (23)	328 (8.6)	26 (4)	12.0 (3.1 to 14.2)	195	167	3	0	2	1	41067	60	5	4	1	42038
SHHEC	UK	1986	1999	10905	49 (7)	5364 (49)	4834 (44)	143 (1.3)	26 (4)	10.0 (6.9 to 10.0)	362	266	1	1	1	0	105279	140	0	0	0	106569
SHIP	Germany	1999	2011	3102	54 (13)	1495 (48)	873 (28)	371 (12.0)	28 (5)	10.4 (0.0 to 12.2)	29	29	0	0	0	0	20768	55	7	6	1	33416
SPEED	UK	1980	1997	2145	55 (4)	2145 (100)	1011 (47)	39 (1.8)	26 (3)	16.7 (3.3 to 18.2)	254	98	5	0	5	0	31239	191	6	5	1	32650
TROMSØ	Norway	1986	2009	14659	39 (10)	7153 (49)	6598 (45)	75 (0.5)	24 (3)	18.9 (11.5 to 19.3)	631	576	2	0	0	2	265073	153	3	1	2	317982
ULSAM	Sweden	1972	2008	1698	50 (1)	1698 (100)	1156 (68)	86 (5.1)	25 (3)	26.5 (6.0 to 37.6)	459	338	70	68	25	45	42188	331	9	7	2	47258
WCWC	Germany	1989	2008	10574	48 (8)	10574 (100)	5051 (48)	631 (6.0)	27 (4)	0.0 (0.0 to 14.6)	28	28	0	0	0	0	37828	193	12	7	5	166503
WHITEI	UK	1997	2010	3995	76 (5)	3995 (100)	554 (14)	184 (4.6)	25 (3)	11.9 (2.0 to 13.3)	336	0	24	0	15	9	39512	336	24	15	9	39512
WHITEII	UK	1987	2004	10141	45 (6)	6773 (67)	2614 (26)	90 (0.9)	25 (4)	17.0 (5.1 to 18.8)	435	357	6	0	4	2	153303	84	6	4	2	174040
ZUTE	Netherlands	1985	2000	760	61 (13)	760 (100)	366 (48)	33 (4.3)	25 (3)	15.1 (2.1 to 40.0)	172	161	1	0	1	0	13836	99	2	2	0	15581
SUBTOTAL	-	1989	2008$	369757	52 (9)	213436 (58)	134916 (36)	12177 (3.3)	26 (4)	12.0 (2.9 to 30.3)	19937	12107	588	70	433	155	5158622	12503	638	500	138	5627694
ARIC	USA	1988	2011	13119	54 (6)	5905 (45)	3661 (28)	1391 (10.6)	27 (5)	22.3 (5.1 to 24.6)	1115	809	17	0	13	4	249619	475	20	14	6	261492
CHA	USA	1971	2003	34250	41 (13)	19894 (58)	14243 (42)	-	26 (4)	32.0 (11.6 to 35.6)	3000	0	70	0	62	8	999109	3000	70	62	8	999109
CHARL	USA	1961	2009	2028	50 (11)	947 (47)	1129 (56)	86 (4.2)	25 (5)	24.7 (3.9 to 48.8)	584	255	14	0	10	4	51312	459	18	14	4	56140
HONOL	USA	1992	1999	2436	78 (4)	2436 (100)	184 (8)	630 (25.9)	23 (3)	6.3 (1.5 to 7.6)	151	110	1	0	1	0	14054	59	1	1	0	14562
HPFS1	USA	1986	2010	45844	54 (10)	45844 (100)	4555 (10)	1280 (2.8)	26 (3)	20.2 (6.2 to 21.9)	2492	0	45	0	41	4	832141	2492	45	41	4	832141
MRFIT	USA	1974	1985	3774	47 (6)	3774 (100)	2330 (62)	108 (2.9)	28 (3)	7.4 (4.9 to 8.0)	224	178	2	0	2	0	26977	110	2	2	0	41381
NHANESI	USA	1972	2000	8209	49 (15)	3120 (38)	2451 (30)	416 (5.1)	26 (5)	19.0 (4.2 to 21.1)	746	277	91	83	26	65	137094	639	14	12	2	140722
NHS1	USA	1976	2009	117852	43 (7)	0 (0)	38875 (33)	2052 (1.7)	24 (4)	28.6 (12.1 to 30.3)	2265	0	104	0	100	4	3138287	2265	104	100	4	3138287
QUEBEC	Canada	1974	2002	2081	46 (8)	2081 (100)	1636 (79)	62 (3.0)	26 (4)	26.4 (4.0 to 26.9)	364	317	5	0	5	0	43348	78	6	6	0	49609
RANCHO	USA	1985	2006	1820	68 (11)	753 (41)	250 (14)	89 (4.9)	25 (4)	14.2 (2.0 to 18.1)	246	243	2	0	1	1	21883	149	2	1	1	23941
SHS	USA	1990	2003	2612	56 (8)	1070 (41)	1063 (41)	1010 (38.7)	30 (6)	12.5 (2.0 to 14.3)	318	227	4	0	4	0	28046	115	6	5	1	29876
WHIOS	USA	1994	2012	81253	63 (7)	0 (0)	4957 (6)	3697 (4.5)	27 (6)	12.0 (4.3 to 14.8)	2492	2101	37	0	37	0	892339	966	62	62	0	912646
SUBTOTAL	-	1981	2005	315278	52 (9)	85824 (27)	75334 (24)	10821 (3.4)	25 (5)	20.4 (6.0 to 32.3)	13997	4517	392	83	302	90	6434208	10807	350	320	30	6499906
AUSDIAB	Australia	2000	2012	8460	52 (12)	3707 (44)	1449 (17)	372 (4.4)	27 (5)	5.1 (4.9 to 13.1)	85	41	1	0	1	0	54771	73	2	2	0	103726
BHS	Australia	1969	2004	5889	45 (16)	2779 (47)	1843 (31)	124 (2.1)	25 (4)	27.0 (7.2 to 33.2)	508	0	12	0	9	3	144381	508	12	9	3	144381
DUBBO	Australia	1989	2003	2054	68 (7)	865 (42)	322 (16)	139 (6.8)	26 (4)	14.1 (1.8 to 14.9)	283	220	7	0	5	2	22798	211	7	5	2	24523
GOH	Israel	1970	2006	2384	43 (8)	1186 (50)	722 (30)	-	25 (4)	35.0 (16.9 to 36.0)	88	0	5	0	3	2	76696	88	5	3	2	76696
HIMS	Australia	1997	2010	8675	72 (4)	8675 (100)	1008 (12)	864 (10.0)	27 (4)	9.1 (0.8 to 13.9)	652	442	151	147	64	87	72360	540	9	5	4	95091
HISAYAMA	Japan	1988	2002	2576	59 (12)	1088 (42)	349 (14)	209 (8.1)	23 (3)	14.0 (3.2 to 14.0)	77	67	2	0	2	0	31587	27	2	2	0	32729
IKNS	Japan	1991	2008	8038	58 (10)	3299 (41)	1911 (24)	556 (6.9)	23 (3)	11.1 (5.1 to 18.6)	84	37	1	0	1	0	91160	58	1	1	0	91435
PRHHP	Caribbean	1967	1980	6342	54 (6)	6342 (100)	3317 (52)	626 (9.9)	25 (4)	8.3 (5.2 to 12.0)	222	135	10	0	4	6	53066	153	13	6	7	71942
TARFS	Turkey	1990	2014	2275	45 (14)	1131 (50)	845 (37)	94 (4.1)	26 (5)	16.6 (4.7 to 17.6)	121	44	0	0	0	0	32171	163	2	2	0	39493
SUBTOTAL	-	1989	2006	46693	64 (11)	29072 (62)	11766 (25)	2984 (6.4)	25 (4)	10.1 (2.7 to 33.1)	2120	986	189	147	89	100	578990	1821	53	35	18	680017
ERFC TOTAL	-	1986	2004	731728	52 (9)	328332 (45)	222016 (30)	25982 (3.6)	25 (4)	14.7 (4.0 to 31.8)	36054	17610	1169	300	824	345	12171819	25131	1041	855	186	12807616
UKBB TOTAL	UK	2009	2016	421537	56 (8)	187838 (45)	43847 (10)	17622 (4.2)	27 (5)	6.1 (4.8 to 7.5)	3385	2808	2321	2234	1273	1048	2566621	802	127	45	82	2947666

* The cohorts are grouped according to 3 geographic locations: Western Europe, North America, and Other.

† Majority of the studies in ERFC did not ascertain nonfatal VTE outcomes; hence analyses in ERFC were restricted to comparison of fatal outcomes only.

**Follow up until first event is used in UKBB; follow up until fatal event is used in the ERFC. The median (5th, 95th) percentiles of fatal events follow up were 15.4 (5.5, 32.0) years in ERFC and 7.0 (5.7, 8.4) years in UKBB.

$ For summary of maximum year of follow up, the SUBTOTAL and TOTAL rows give the median value across studies in ERFC.

© 2019 Gregson J et al. *JAMA Cardiol.*

eTable 2. Hazard ratios for VTE per 1-SD higher usual risk factor value or group contrast, with and without adjustment for BMI*

			Adjusted for age, sex, smoking status, history of diabetes		Adjusted for age, sex, smoking status, history of diabetes, **and BMI**	
Variable	N	Events	HR (95% CI)	P-value	HR (95% CI)	P-value
**UK Biobank (VTE)**						
Age (yrs): Per decade	421537	2321	1.76 (1.66, 1.86)	<0.0001	1.81 (1.71, 1.92)	<0.0001
Sex (male vs. female)	421537	2321	1.45 (1.34, 1.58)	<0.0001	1.44 (1.32, 1.56)	<0.0001
Smoking (current vs. other)	421537	2321	1.23 (1.08, 1.40)	0.002	1.23 (1.08, 1.40)	0.002
Diabetes (yes vs. no)	421537	2321	1.08 (0.90, 1.30)	0.395	0.83 (0.69, 0.99)	0.041
Alcohol (current vs. other)	421197	2319	0.77 (0.67, 0.89)	<0.001	0.82 (0.71, 0.94)	0.006
Systolic blood pressure (mmHg)	421179	2315	0.93 (0.86, 1.00)	0.056	0.83 (0.77, 0.90)	<0.0001
Diastolic blood pressure (mmHg)	421181	2315	1.12 (1.04, 1.21)	0.004	0.94 (0.87, 1.02)	0.150
Body mass index (kg/m^2)	421537	2321	1.37 (1.32, 1.41)	<0.0001	1.37 (1.32, 1.41)	<0.0001
Waist:Hip ratio	421440	2319	1.52 (1.40, 1.65)	<0.0001	1.13 (1.02, 1.25)	0.025
Waist circumference (cm)	421464	2320	1.60 (1.52, 1.68)	<0.0001	1.68 (1.44, 1.97)	<0.0001
**ERFC (Fatal VTE)**						
Age (yrs): Per decade	731728	1041	2.58 (2.38, 2.81)	<0.0001	2.67 (2.45, 2.91)	<0.0001
Sex (male vs. female)	731728	1041	1.15 (0.98, 1.35)	0.094	1.17 (1.00, 1.38)	0.053
Smoking (current vs. other)	731728	1041	1.28 (1.12, 1.47)	<0.001	1.38 (1.20, 1.58)	<0.0001
Diabetes (yes vs. no)	731728	1041	1.93 (1.51, 2.46)	<0.0001	1.69 (1.33, 2.16)	<0.0001
Alcohol (current vs. other)	386831	564	0.74 (0.59, 0.91)	0.005	0.75 (0.61, 0.93)	0.010
Systolic blood pressure (mmHg)	566724	887	1.17 (1.04, 1.30)	0.006	1.07 (0.95, 1.19)	0.277
Diastolic blood pressure (mmHg)	565895	885	1.41 (1.25, 1.59)	<0.0001	1.26 (1.11, 1.42)	<0.001
Body mass index (kg/m^2)	731728	1041	1.43 (1.35, 1.50)	<0.0001	1.43 (1.35, 1.50)	<0.0001
Waist:Hip ratio	264787	329	1.23 (1.05, 1.45)	0.012	1.01 (0.82, 1.25)	0.915
Waist circumference (cm)	265465	333	1.54 (1.37, 1.73)	<0.0001	1.35 (1.04, 1.75)	0.026
Total cholesterol (mmol/l)	455177	732	1.01 (0.89, 1.13)	0.930	0.99 (0.88, 1.11)	0.838
Non HDL cholesterol (mmol/l)	311888	440	0.92 (0.79, 1.07)	0.290	0.88 (0.76, 1.03)	0.107
HDL cholesterol (mmol/l)	312207	440	0.98 (0.85, 1.13)	0.795	1.09 (0.94, 1.26)	0.272
Log Triglycerides (mmol/l)	322096	537	1.08 (0.93, 1.25)	0.307	0.95 (0.81, 1.11)	0.485
Apolipoprotein B (g/l)	80712	261	0.80 (0.65, 0.98)	0.035	0.76 (0.61, 0.94)	0.011
Apolipoprotein A1 (g/l)	84483	265	0.60 (0.44, 0.80)	0.001	0.65 (0.47, 0.88)	0.006
Log Lp(a) (mg/dl)	66382	210	0.83 (0.71, 0.98)	0.030	0.83 (0.71, 0.98)	0.030
Fasting glucose (mmol/l)	130322	323	1.31 (1.13, 1.52)	<0.001	1.27 (1.08, 1.48)	0.003
Log CRP (mg/l)	70855	231	1.82 (1.46, 2.28)	<0.0001	1.71 (1.33, 2.20)	<0.0001
Fibrinogen (µmol/l)	115002	223	1.42 (1.16, 1.73)	0.001	1.37 (1.11, 1.68)	0.003
Albumin (g/l)	115309	367	0.74 (0.55, 1.00)	0.052	0.75 (0.56, 1.02)	0.066

* Associations involve ERFC data for fatal VTE and UK Biobank data for VTE.

© 2019 Gregson J et al. *JAMA Cardiol.*

eFigure 1. Hazard ratios for fatal/nonfatal VTE by alcohol consumption among current drinkers in UK Biobank

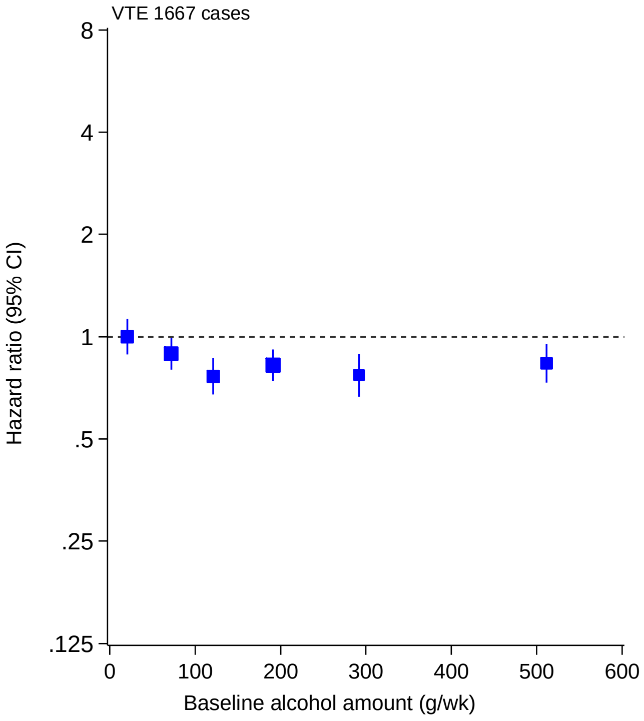

VTE, venous thromboembolism.

*Adjusted for age, sex, smoking status, history of diabetes, and BMI. The 6 categories of baseline alcohol consumption were 0 to ≤50 (Reference), 50 to ≤100, 100 to ≤150, 150 to ≤250, 250 to ≤350, and >350 g/wk. Hazard ratios are plotted against the mean baseline alcohol consumption in each category. Sizes of the boxes are proportional to the inverse of the variance of the log-transformed hazard ratios. Vertical lines represent 95% CIs.

© 2019 Gregson J et al. *JAMA Cardiol.*

eFigure 2. Hazard ratios for nonfatal vs fatal VTE per 1-SD higher usual risk factor value or group contrast, with and without adjustment for BMI

UK Biobank (with BMI adjustment)

Exposure \ Outcome	N	Events	HR (95% CI)	Association P-value
**Age (yrs): Per decade**				
Nonfatal VTE	421537	2234	1.79 (1.69, 1.89)	<0.0001
Fatal VTE	421537	87	2.74 (1.97, 3.82)	<0.0001
**Sex (male vs. female)**				
Nonfatal VTE	421537	2234	1.45 (1.33, 1.57)	<0.0001
Fatal VTE	421537	87	1.20 (0.78, 1.83)	0.402
**Smoking (current vs. other)**				
Nonfatal VTE	421537	2234	1.21 (1.06, 1.38)	0.005
Fatal VTE	421537	87	1.83 (1.01, 3.32)	0.046
**Diabetes (yes vs. no)**				
Nonfatal VTE	421537	2234	0.76 (0.62, 0.92)	0.005
Fatal VTE	421537	87	2.44 (1.36, 4.39)	0.003
**Alcohol (current vs. other)**				
Nonfatal VTE	421197	2232	0.84 (0.73, 0.97)	0.020
Fatal VTE	421197	87	0.51 (0.29, 0.92)	0.025
**Systolic blood pressure (mmHg)**				
Nonfatal VTE	421179	2228	0.82 (0.76, 0.89)	<0.0001
Fatal VTE	421179	87	1.28 (0.88, 1.88)	0.197
**Diastolic blood pressure (mmHg)**				
Nonfatal VTE	421181	2228	0.93 (0.85, 1.01)	0.082
Fatal VTE	421181	87	1.39 (0.92, 2.10)	0.117
**Body mass index (kg/m^2)**				
Nonfatal VTE	421537	2234	1.36 (1.31, 1.40)	<0.0001
Fatal VTE	421537	87	1.63 (1.41, 1.90)	<0.0001
**Waist:Hip ratio**				
Nonfatal VTE	421440	2232	1.49 (1.38, 1.62)	<0.0001
Fatal VTE	421440	87	2.25 (1.62, 3.11)	<0.0001
**Waist circumference (cm)**				
Nonfatal VTE	421464	2233	1.58 (1.50, 1.66)	<0.0001
Fatal VTE	421464	87	2.16 (1.72, 2.72)	<0.0001

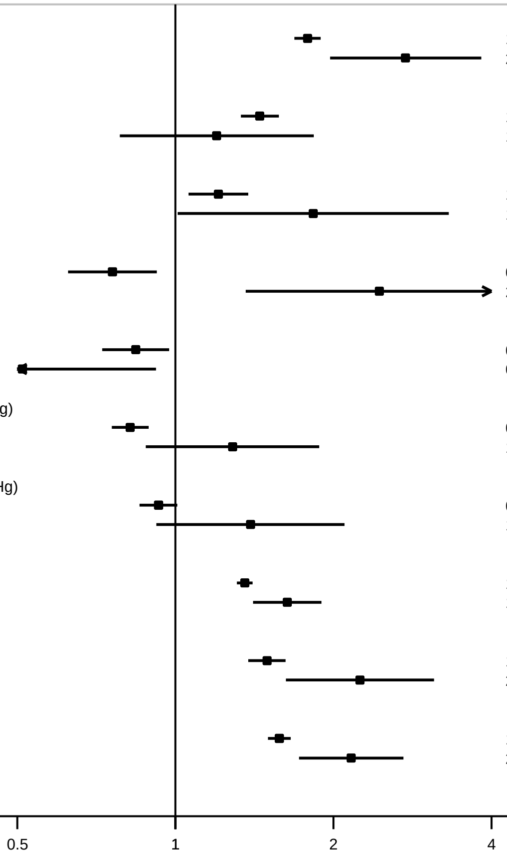

UK Biobank (without BMI adjustment)

Exposure \ Outcome	N	Events	HR (95% CI)	Association P-value
**Age (yrs): Per decade**				
Nonfatal VTE	421537	2234	1.74 (1.64, 1.84)	<0.0001
Fatal VTE	421537	87	2.53 (1.83, 3.50)	<0.0001
**Sex (male vs. female)**				
Nonfatal VTE	421537	2234	1.46 (1.35, 1.59)	<0.0001
Fatal VTE	421537	87	1.14 (0.75, 1.75)	0.534
**Smoking (current vs. other)**				
Nonfatal VTE	421537	2234	1.21 (1.06, 1.38)	0.005
Fatal VTE	421537	87	1.82 (1.01, 3.30)	0.047
**Diabetes (yes vs. no)**				
Nonfatal VTE	421537	2234	0.99 (0.81, 1.19)	0.884
Fatal VTE	421537	87	3.86 (2.19, 6.79)	<0.0001
**Alcohol (current vs. other)**				
Nonfatal VTE	421197	2232	0.80 (0.69, 0.92)	0.002
Fatal VTE	421197	87	0.46 (0.26, 0.82)	0.009
**Systolic blood pressure (mmHg)**				
Nonfatal VTE	421179	2228	0.91 (0.84, 0.99)	0.020
Fatal VTE	421179	87	1.43 (1.00, 2.06)	0.052
**Diastolic blood pressure (mmHg)**				
Nonfatal VTE	421181	2228	1.10 (1.02, 1.19)	0.018
Fatal VTE	421181	87	1.73 (1.18, 2.55)	0.005
**Body mass index (kg/m^2)**				
Nonfatal VTE	421537	2234	1.36 (1.31, 1.40)	<0.0001
Fatal VTE	421537	87	1.63 (1.41, 1.90)	<0.0001
**Waist:Hip ratio**				
Nonfatal VTE	421440	2232	1.49 (1.38, 1.62)	<0.0001
Fatal VTE	421440	87	2.25 (1.62, 3.11)	<0.0001
**Waist circumference (cm)**				
Nonfatal VTE	421464	2233	1.58 (1.50, 1.66)	<0.0001
Fatal VTE	421464	87	2.16 (1.72, 2.72)	<0.0001

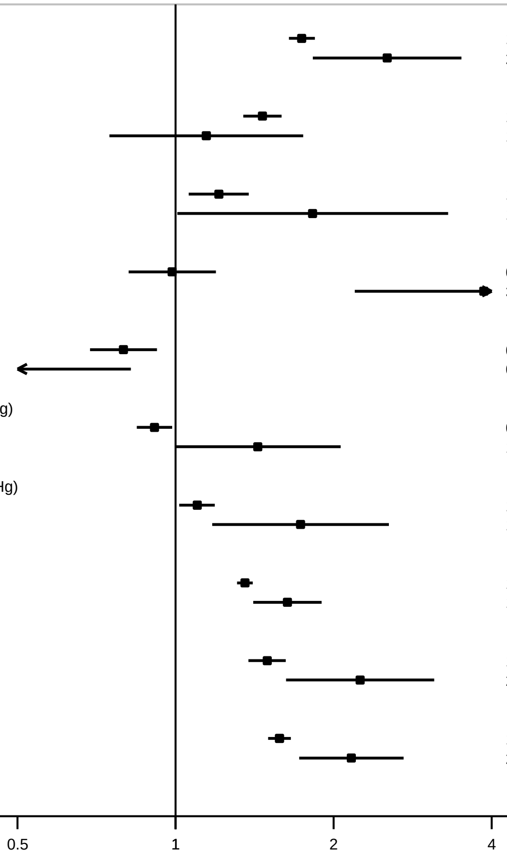

VTE, venous thromboembolism;

*Left figure: Adjusted for age, sex, smoking status, history of diabetes, and baseline BMI (except for WHR and waist that were not adjusted for baseline BMI); Right figure: Adjusted for age, sex, smoking status, and history of diabetes. Associations involve UK Biobank data only.

© 2019 Gregson J et al. *JAMA Cardiol.*

eFigure 3. Hazard ratios for fatal CHD vs fatal VTE (left) and fatal/nonfatal CHD vs VTE (right) per 1-SD higher usual risk factor value or group contrast

ERFC

Exposure \ Outcome	N	Events	HR (95% CI)	Difference P-value
Age (yrs): Per decade				
Fatal CHD	731728	25131	3.23 (3.18, 3.29)	<0.0001
Fatal VTE	731728	1041	2.67 (2.45, 2.91)	
Sex (male vs. female)				
Fatal CHD	731728	25131	2.06 (1.99, 2.14)	<0.0001
Fatal VTE	731728	1041	1.17 (1.00, 1.38)	
Smoking (current vs. other)				
Fatal CHD	731728	25131	2.06 (2.00, 2.12)	<0.0001
Fatal VTE	731728	1041	1.38 (1.20, 1.58)	
Diabetes (yes vs. no)				
Fatal CHD	731728	25131	2.74 (2.62, 2.87)	<0.001
Fatal VTE	731728	1041	1.69 (1.33, 2.16)	
Alcohol (current vs. other)				
Fatal CHD	386831	10844	0.79 (0.75, 0.83)	0.711
Fatal VTE	386831	564	0.75 (0.61, 0.93)	
Systolic blood pressure (mmHg)				
Fatal CHD	566724	20310	1.65 (1.61, 1.68)	<0.0001
Fatal VTE	566724	887	1.07 (0.95, 1.19)	
Diastolic blood pressure (mmHg)				
Fatal CHD	565895	20210	1.56 (1.52, 1.60)	0.001
Fatal VTE	565895	885	1.25 (1.11, 1.42)	
Body mass index (kg/m^2)				
Fatal CHD	731728	25131	1.27 (1.25, 1.28)	<0.0001
Fatal VTE	731728	1041	1.43 (1.35, 1.50)	
Waist:Hip ratio				
Fatal CHD	264787	5229	1.37 (1.32, 1.42)	0.223
Fatal VTE	264787	329	1.23 (1.05, 1.45)	
Waist circumference (cm)				
Fatal CHD	265465	5325	1.29 (1.25, 1.33)	0.003
Fatal VTE	265465	333	1.54 (1.37, 1.73)	

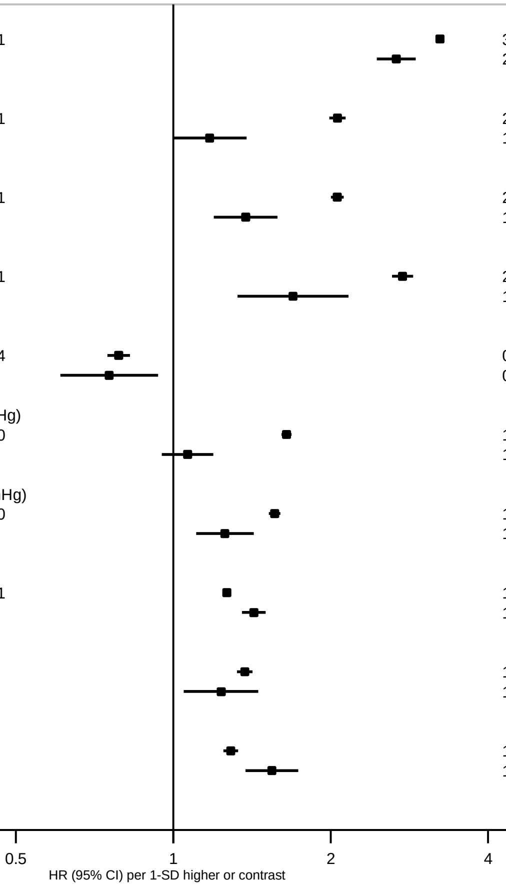

UK Biobank

Exposure \ Outcome	N	Events	HR (95% CI)	Difference P-value
Age (yrs): Per decade				
CHD	421537	3385	1.97 (1.88, 2.07)	0.023
VTE	421537	2321	1.81 (1.71, 1.92)	
Sex (male vs. female)				
CHD	421537	3385	3.64 (3.37, 3.94)	<0.0001
VTE	421537	2321	1.44 (1.32, 1.56)	
Smoking (current vs. other)				
CHD	421537	3385	2.59 (2.39, 2.81)	<0.0001
VTE	421537	2321	1.23 (1.08, 1.40)	
Diabetes (yes vs. no)				
CHD	421537	3385	1.40 (1.24, 1.58)	<0.0001
VTE	421537	2321	0.83 (0.69, 0.99)	
Alcohol (current vs. other)				
CHD	421197	3379	0.71 (0.63, 0.80)	0.139
VTE	421197	2319	0.82 (0.71, 0.94)	
Systolic blood pressure (mmHg)				
CHD	421179	3383	1.60 (1.50, 1.70)	<0.0001
VTE	421179	2315	0.83 (0.77, 0.90)	
Diastolic blood pressure (mmHg)				
CHD	421181	3383	1.52 (1.42, 1.62)	<0.0001
VTE	421181	2315	0.94 (0.87, 1.02)	
Body mass index (kg/m^2)				
CHD	421537	3385	1.18 (1.14, 1.21)	<0.0001
VTE	421537	2321	1.37 (1.32, 1.41)	
Waist:Hip ratio				
CHD	421440	3384	1.59 (1.49, 1.70)	0.425
VTE	421440	2319	1.52 (1.40, 1.65)	
Waist circumference (cm)				
CHD	421464	3384	1.27 (1.22, 1.33)	<0.0001
VTE	421464	2320	1.60 (1.52, 1.68)	

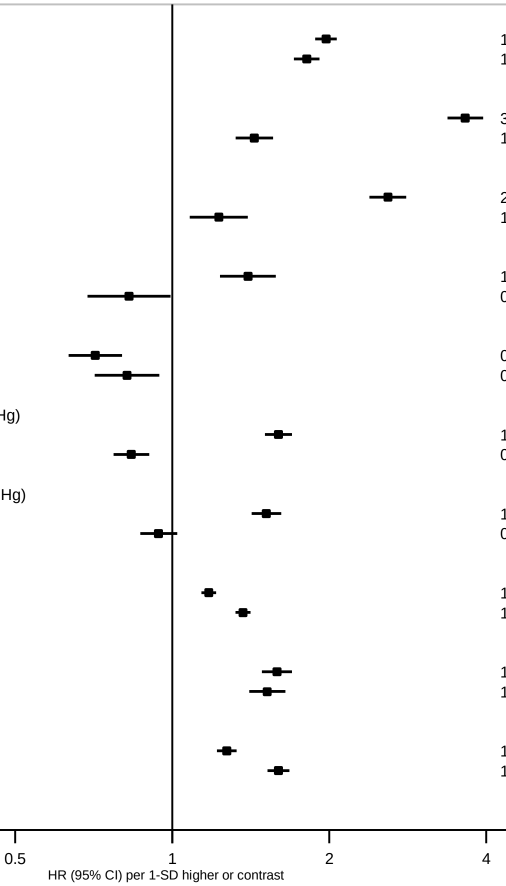

ERFC, Emerging Risk Factors Collaboration; VTE, venous thromboembolism; CHD, coronary heart disease.

Adjusted for age, sex, smoking status, history of diabetes, and usual BMI (except for WHR and waist that were not adjusted for usual BMI).
*The difference p-value indicates overall evidence for difference in hazard ratios for VTE vs. CHD based on fixed effect meta-analysis of the ratio of hazard ratios for fatal VTE vs. Fatal CHD (left figure) and VTE vs. CHD (right figure) on the log-scale.

© 2019 Gregson J et al. *JAMA Cardiol.*

eFigure 4. Hazard ratios for fatal CHD vs fatal VTE per 1-SD higher usual levels of biochemical markers

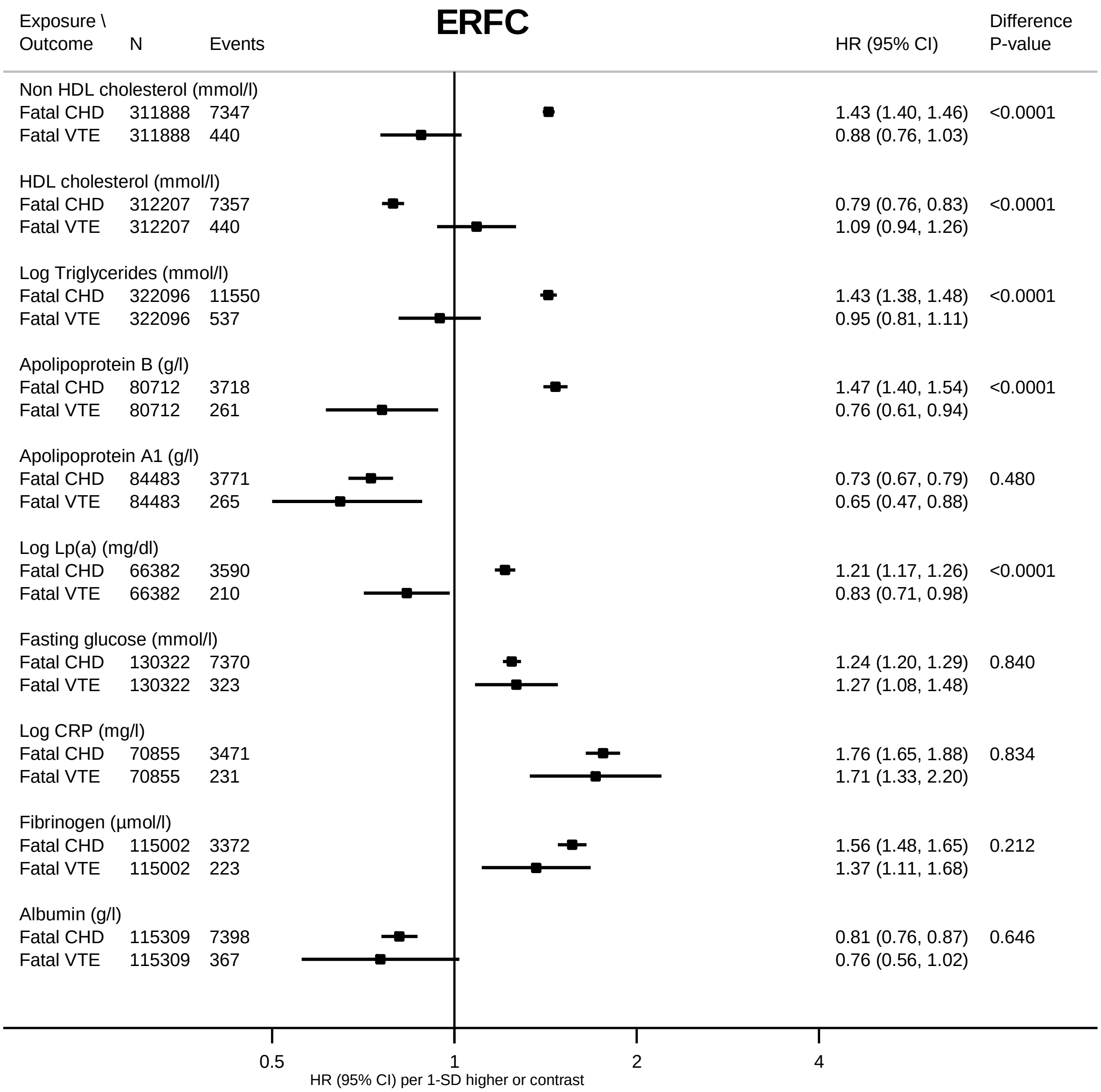

ERFC, Emerging Risk Factors Collaboration; HDL-C, high density lipoprotein cholesterol; CRP, C-reactive protein; Lp(a), lipoprotein(a); VTE, venous thromboembolism; CHD, coronary heart disease.
*Adjusted for age, sex, smoking status, history of diabetes, and baseline BMI. Associations involve ERFC data only.

© 2019 Gregson J et al. *JAMA Cardiol.*

eFigure 5. Hazard ratios for unprovoked and provoked VTE per 1-SD higher usual risk factor value or group contrast in UK Biobank after excluding participants with history of cancer

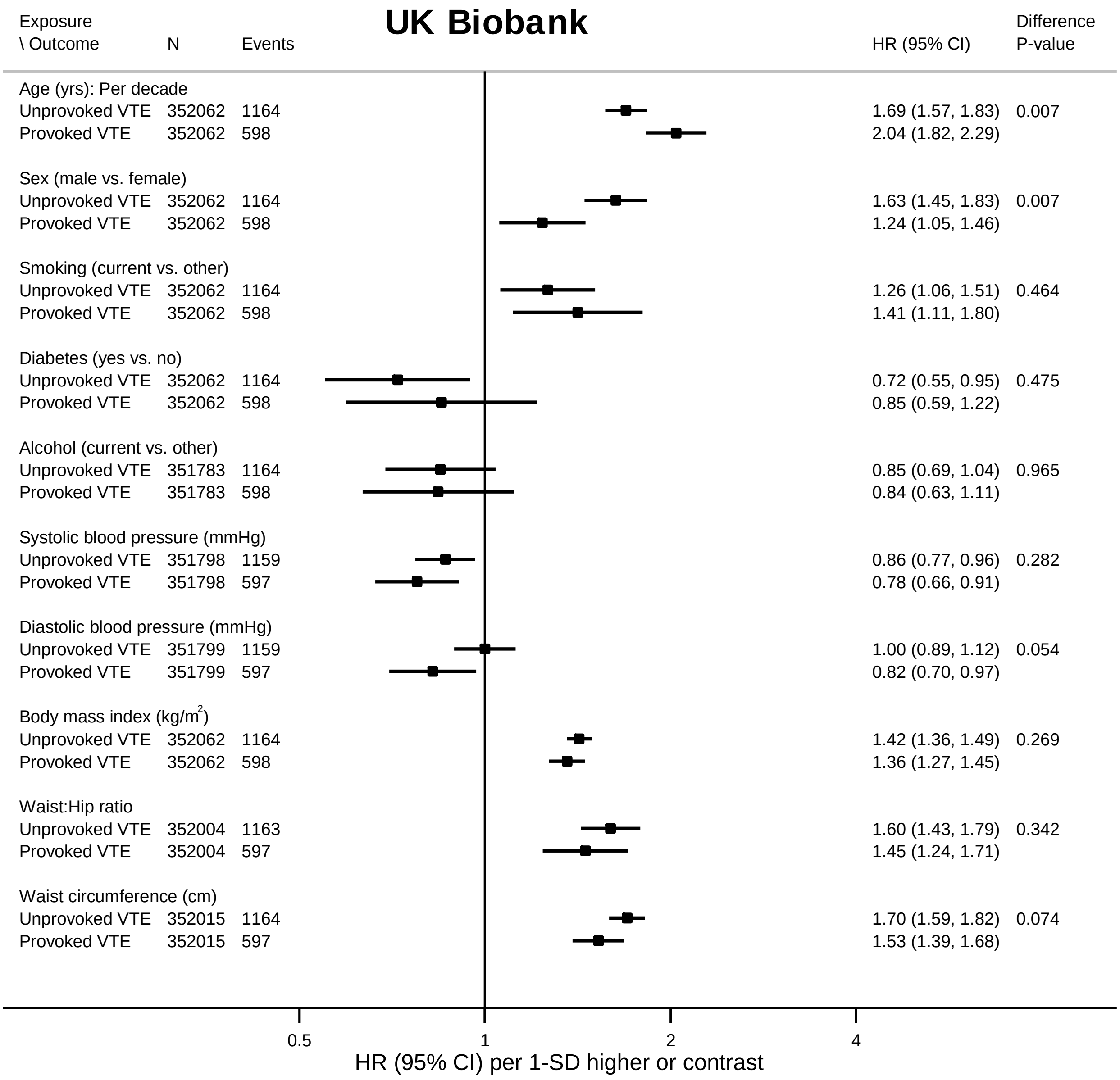

VTE, venous thromboembolism.

*Adjusted for age, sex, smoking status, history of diabetes, and usual BMI (except for WHR and waist that were not adjusted for usual BMI). Associations involve UK Biobank data only.

© 2019 Gregson J et al. *JAMA Cardiol.*

eFigure 6. Hazard ratios for fatal CHD vs fatal VTE per 1-SD higher usual risk factor value or group contrast, with censoring at first event

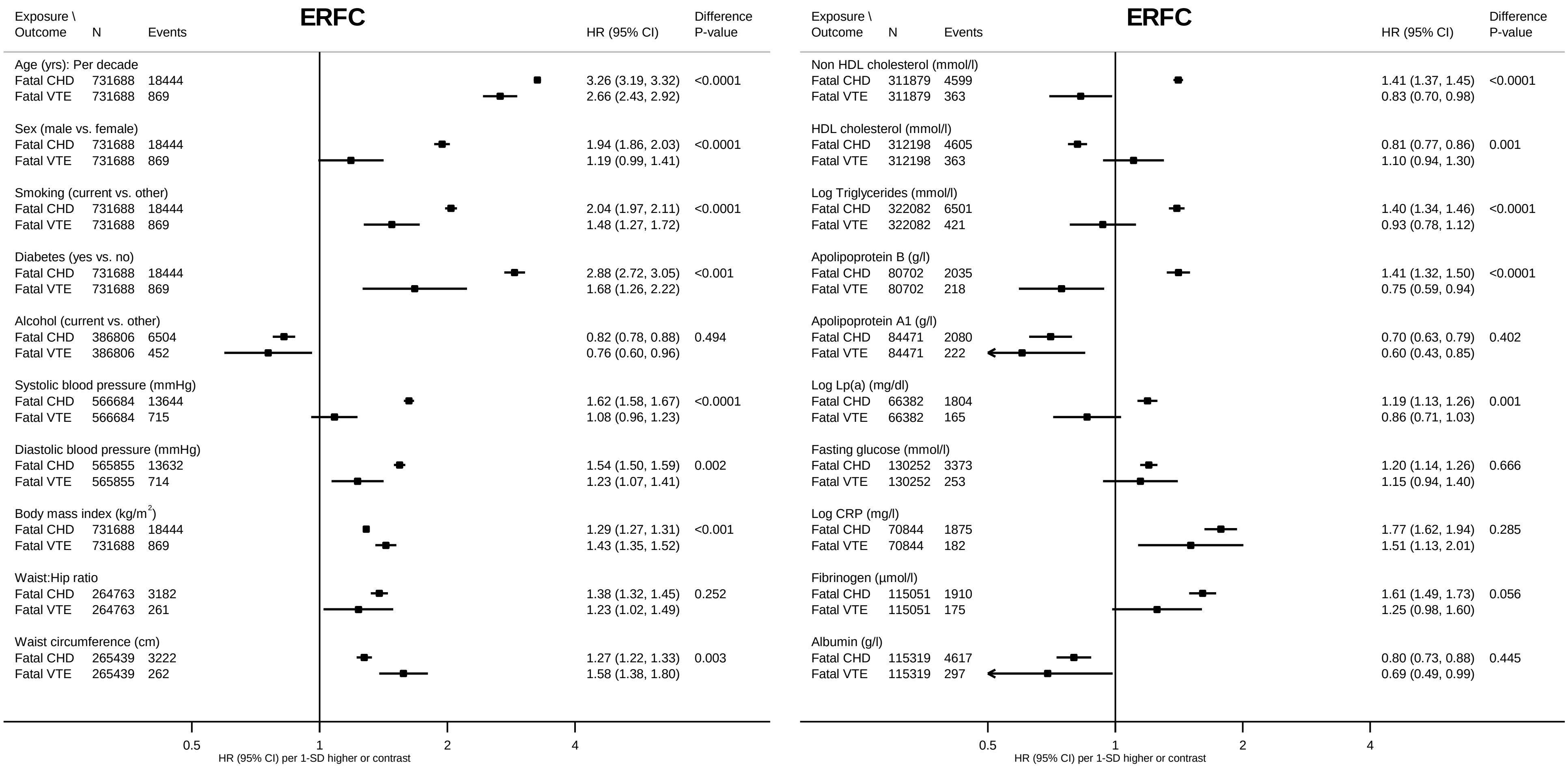

ERFC, Emerging Risk Factors Collaboration; VTE, venous thromboembolism; CHD, coronary heart disease.
*Adjusted for age, sex, smoking status, history of diabetes, and baseline BMI (except for WHR and waist that were not adjusted for baseline BMI).

© 2019 Gregson J et al. *JAMA Cardiol.*

eFigure 7. Hazard ratios for VTE per 1-SD higher baseline risk factor value or group contrast

Exposure \ Outcome	N	Events	HR (95% CI)	Association P-value
**Age (yrs): Per decade**				
UKBB	421537	2321	1.78 (1.68, 1.89)	<0.0001
ERFC	731728	1041	2.58 (2.37, 2.80)	<0.0001
**Sex (male vs. female)**				
UKBB	421537	2321	1.44 (1.33, 1.56)	<0.0001
ERFC	731728	1041	1.15 (0.98, 1.35)	0.096
**Smoking (current vs. other)**				
UKBB	421537	2321	1.27 (1.12, 1.45)	<0.001
ERFC	731728	1041	1.39 (1.21, 1.60)	<0.0001
**Diabetes (yes vs. no)**				
UKBB	421537	2321	0.81 (0.67, 0.97)	0.025
ERFC	731728	1041	1.63 (1.27, 2.08)	<0.0001
**Alcohol (current vs. other)**				
UKBB	421197	2319	0.82 (0.71, 0.94)	0.005
ERFC	386831	564	0.75 (0.61, 0.93)	0.009
**Systolic blood pressure (mmHg)**				
UKBB	421179	2315	0.89 (0.85, 0.93)	<0.0001
ERFC	566724	887	1.03 (0.96, 1.10)	0.460
**Diastolic blood pressure (mmHg)**				
UKBB	421181	2315	0.96 (0.91, 1.00)	0.079
ERFC	565895	885	1.11 (1.04, 1.18)	0.003
**Body mass index (kg/m^2)**				
UKBB	421537	2321	1.34 (1.30, 1.38)	<0.0001
ERFC	731728	1041	1.40 (1.33, 1.47)	<0.0001
**Waist:Hip ratio**				
UKBB	421440	2319	1.29 (1.23, 1.36)	<0.0001
ERFC	264787	329	1.15 (1.03, 1.28)	0.015
**Waist circumference (cm)**				
UKBB	421464	2320	1.47 (1.41, 1.53)	<0.0001
ERFC	265465	333	1.46 (1.32, 1.62)	<0.0001
**Non HDL cholesterol (mmol/l)**				
ERFC	311888	440	0.92 (0.83, 1.01)	0.082
**HDL cholesterol (mmol/l)**				
ERFC	312207	440	1.06 (0.95, 1.17)	0.295
**Log Triglycerides (mmol/l)**				
ERFC	322096	537	0.99 (0.90, 1.09)	0.853
**Apolipoprotein B (g/l)**				
ERFC	80712	261	0.86 (0.76, 0.98)	0.026
**Apolipoprotein A1 (g/l)**				
ERFC	84483	265	0.87 (0.77, 0.98)	0.022
**Log Lp(a) (mg/dl)**				
ERFC	66382	210	0.88 (0.77, 1.00)	0.052
**Fasting glucose (mmol/l)**				
ERFC	130322	323	1.14 (1.04, 1.26)	0.008
**Log CRP (mg/l)**				
ERFC	70855	231	1.36 (1.18, 1.56)	<0.0001
**Fibrinogen (µmol/l)**				
ERFC	115002	223	1.18 (1.05, 1.32)	0.004
**Albumin (g/l)**				
ERFC	115309	367	0.86 (0.77, 0.96)	0.005

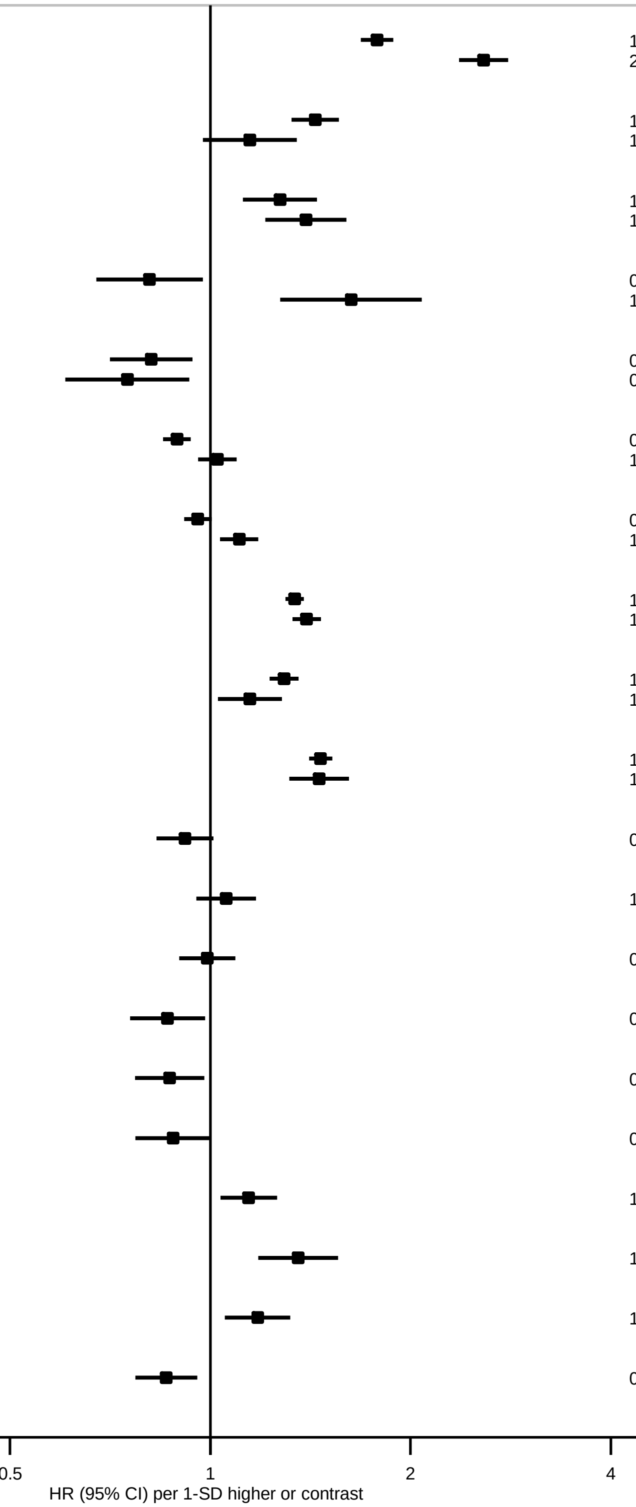

HDL-C, high density lipoprotein cholesterol; CRP, C-reactive protein; Lp(a), lipoprotein(a); VTE, venous thromboembolism.

*Adjusted for age, sex, smoking status, history of diabetes, and baseline BMI (except for WHR and waist that were not adjusted for baseline BMI).

© 2019 Gregson J et al. *JAMA Cardiol.*

eFigure 8. Hazard ratios for fatal CHD vs fatal VTE (left) and fatal/nonfatal CHD vs VTE (right) per 1-SD higher baseline risk factor value or group contrast

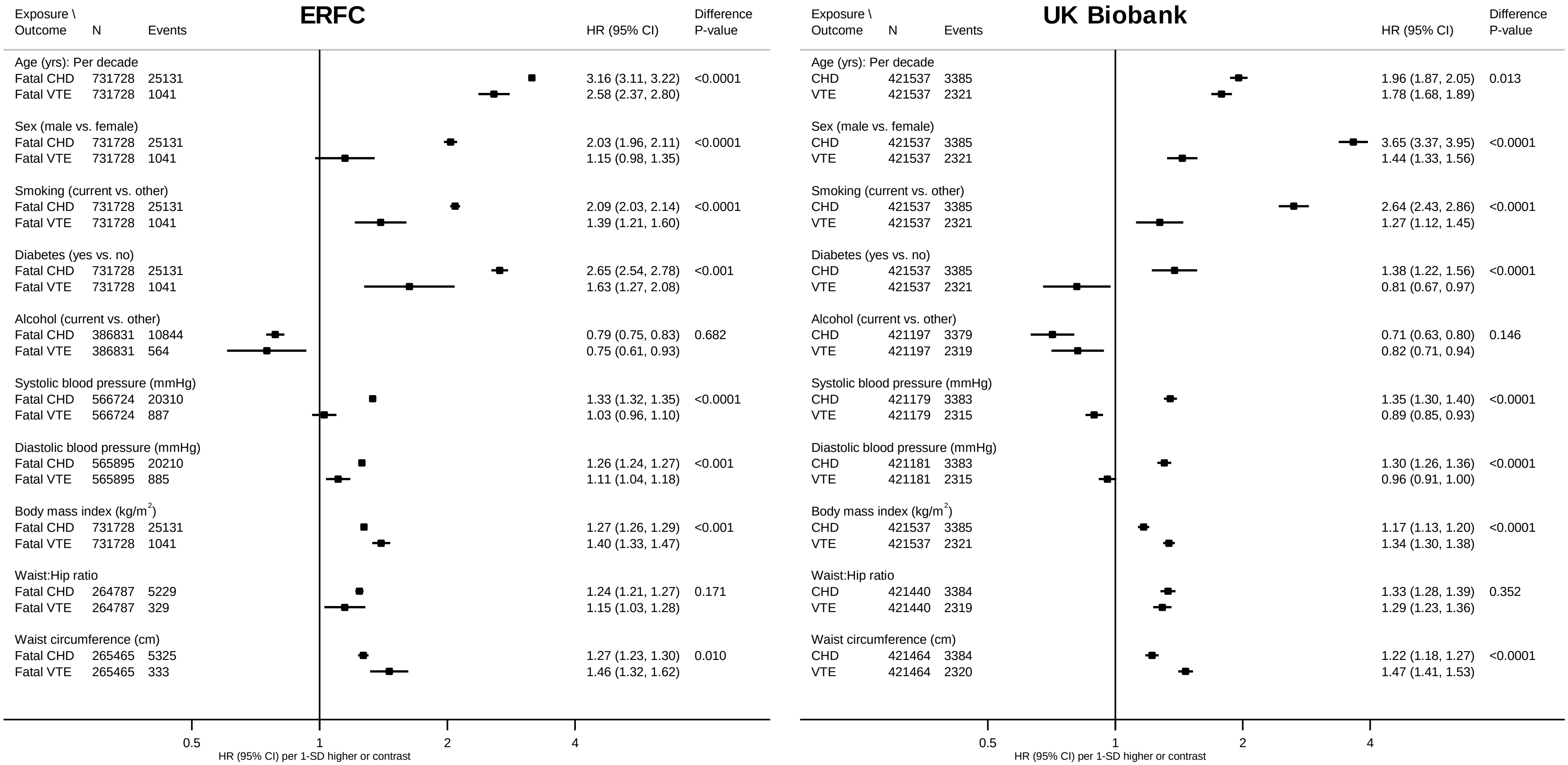

ERFC, Emerging Risk Factors Collaboration; VTE, venous thromboembolism; CHD, coronary heart disease.
*Adjusted for age, sex, smoking status, history of diabetes, and baseline BMI (except for WHR and waist that were not adjusted for baseline BMI).

© 2019 Gregson J et al. *JAMA Cardiol.*

eFigure 9. Hazard ratios for fatal CHD vs fatal VTE per 1-SD higher baseline levels of biochemical markers

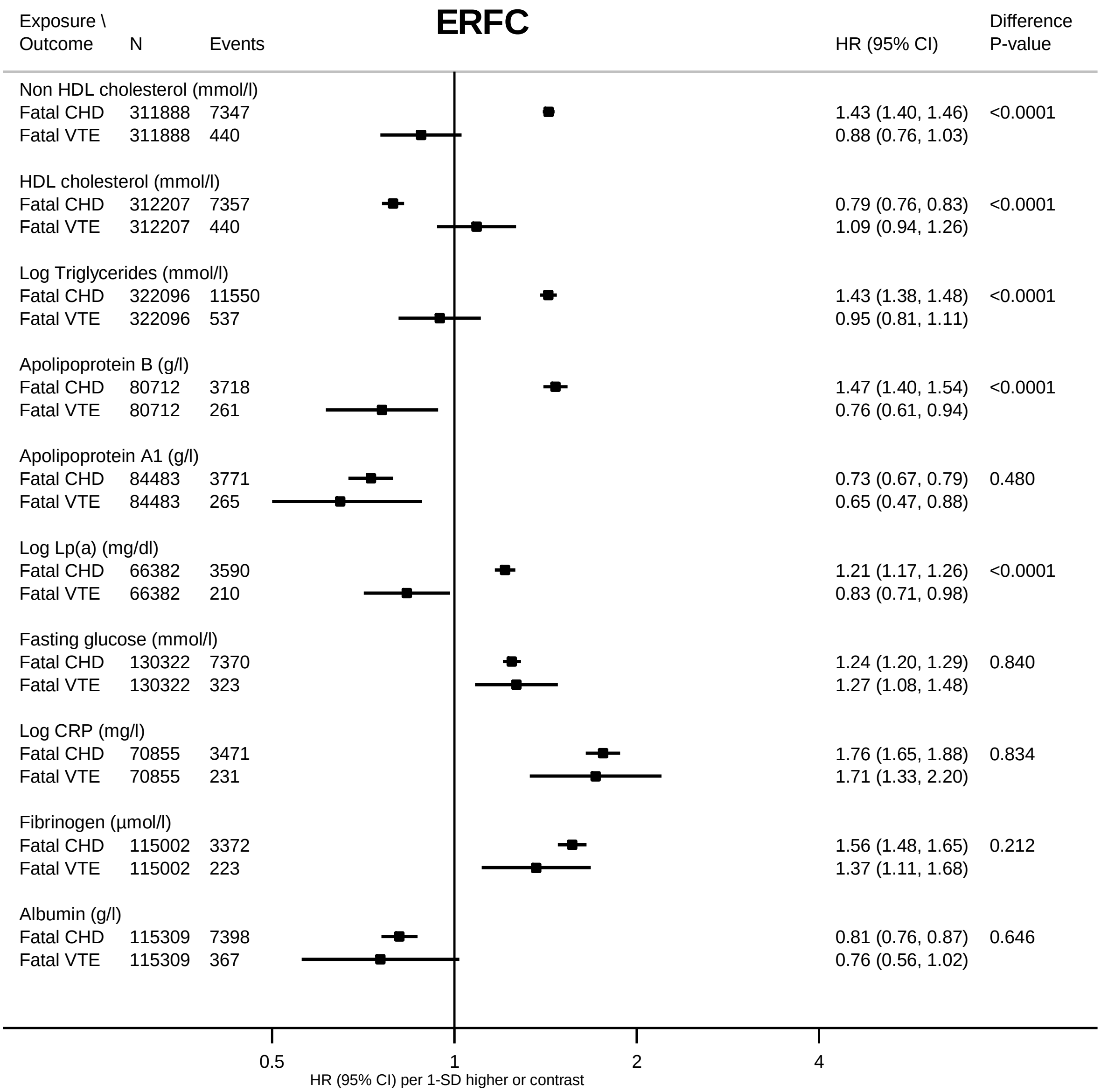

ERFC, Emerging Risk Factors Collaboration; HDL-C, high density lipoprotein cholesterol; CRP, C-reactive protein; Lp(a), lipoprotein(a); VTE, venous thromboembolism; CHD, coronary heart disease.
*Adjusted for age, sex, smoking status, history of diabetes, and baseline BMI. Associations involve ERFC data only.

© 2019 Gregson J et al. *JAMA Cardiol.*
